# Extracellular vesicles as modifiers of antibody‐drug conjugate efficacy

**DOI:** 10.1002/jev2.12070

**Published:** 2021-02-13

**Authors:** Mark Barok, Maija Puhka, Narjes Yazdi, Heikki Joensuu

**Affiliations:** ^1^ Helsinki University Hospital and University of Helsinki Helsinki Finland; ^2^ Laboratory of Molecular Oncology University of Helsinki Biomedicum Helsinki Finland; ^3^ Institute for Molecular Medicine FIMM EV and HiPrep Core University of Helsinki Helsinki Finland

**Keywords:** antibody‐drug conjugate, anti‐cancer drug, drug resistance, extracellular vesicle

## Abstract

Antibody‐drug conjugates (ADCs) are a new class of anti‐cancer drugs that consist of a monoclonal antibody, a highly potent small‐molecule cytotoxic drug, and a chemical linker between the two. ADCs can selectively deliver cytotoxic drugs to cancer cells leading to a reduced systemic exposure and a wider therapeutic window. To date, nine ADCs have received marketing approval, and over 100 are being investigated in nearly 600 clinical trials. The target antigens of at least eight out of the nine approved anti‐cancer ADCs and of 69 investigational ADCs are present on extracellular vesicles (EVs) (tiny particles produced by almost all types of cells) that may carry their contents into local and distant cells. Therefore, the EVs have a potential to mediate both the anti‐cancer effects and the adverse effects of ADCs. In this overview, we discuss the mechanisms of action of ADCs and the resistance mechanisms to them, the EV‐mediated resistance mechanisms to small molecule anti‐cancer drugs and anti‐cancer monoclonal antibodies, and the EVs as modifiers of ADC efficacy and safety.

**Abbreviations**
ABC: ATP binding cassette
ADC: Antibody‐drug conjugate
ADCC: Antibody‐dependent cellular cytotoxicity
AML: Acute myeloid leukaemia
DAR: Drug‐antibody ratio
DM1: Derivative of maytansine 1
EGFR: Epidermal growth factor receptor
EGFRvIII: Epidermal growth factor receptor variant III
EV: Extracellular vesicle
FDA: US Food and Drug Administration
HER2: Human epidermal growth factor receptor‐2
mAb: Monoclonal antibody
MMAE: Monomethyl auristatin E
Trop‐2: Trophoblast cell‐surface antigen 2

## 1 INTRODUCTION

The standard chemotherapy agents often have a relatively narrow therapeutic window (Khongorzul et al., 2020). Antibody‐drug conjugates (ADC) are a novel class of anti‐cancer therapeutics that allow administration of highly cytotoxic drugs to cancer patients utilizing the excellent targeting ability of monoclonal antibodies and their often favourable pharmacokinetic profiles (Beck et al., 2017). The ADC technology may lead to selective delivery of potent small‐molecule cytotoxic drugs to cancer cells while mitigating the adverse effects on non‐malignant cells thus potentially broadening the therapeutic window. Yet, achieving this goal has often remained a challenge (Khongorzul et al., 2020), and the development of many ADCs has been discontinued in the clinical phase due to a poor therapeutic index (Coats et al., 2019).

Besides the cancer cells, the target antigens of ADCs are often expressed to some extent on extracellular vesicles (EVs) (Supplementary Table 1). EVs are small particles surrounded by a lipid membrane bilayer. They are generated from cells and have variable contents. EVs can be carried in the body fluids for long distances, and when they fuse with the target cell, they discard their biologically active contents into the recipient cell potentially changing its function (Al‐Nedawi et al., 2009; Puhka et al., 2017; Ratajczak et al., 2006; Valadi et al., 2007; Van Niel et al., 2018). Since the ADCs may bind to the EVs, and be carried on the EVs (Barok et al., 2018; Hansen et al., 2016), the ADC toxic payload may be liberated even far from the tumour site, thus the EVs could influence both the efficacy and the safety of ADCs.

In this review, we discuss how the EVs can act as modifiers of ADC efficacy and safety. To the best of our knowledge, reviews on this topic are absent in the literature.

## 2 ANTIBODY‐DRUG CONJUGATES (ADCs)

ADCs may be regarded an example of Paul Ehrlich's old idea of a ‘*magic bullet*’ to treat disease using an agent with high efficacy and high specificity (Strebhardt & Ullrich, 2008). Although the ADC era might be just beginning, already nine ADCs have been approved by the US Food and Drug Administration (FDA), five of them for haematological malignancies and four for solid tumours (Table 1). Approximately 100 ADCs targeting over 50 different antigens are currently in the clinical development in nearly 600 clinical trials (Chau et al., 2019; Coats et al., 2019; Khongorzul et al., 2020).

**TABLE 1:** The FDA approved antibody‐drug conjugates (ADCs)

Antibody‐drug conjugate (ADC)	Molecular target	Indications (the year of the FDA approval)	ADC main parts 1) Monoclonal antibody 2) Linker 3) Payload	Drug‐antibody ratio (DAR)	Antitumor effect of the antibody part per se	ATP binding cassette (ABC) drug efflux transporter‐related resistance	Drug resistance reported in patients	Target antigen found on extra‐cellular vesicles
ADCs for haematological malignancies								
Gemtuzumab ozogamicin*	CD33	1) Newly‐diagnosed CD33‐positive acute adult myeloid leukaemia** (2017) (Jen et al., 2018) 2) Relapsed or refractory CD33‐positive acute myeloid leukaemia in adults and in pediatric patients older than 2 years (2017) (Norsworthy et al., 2018)	1) Humanized anti‐CD33 2) pH‐sensitive butanoic acid 3) Calicheamicin (Ciravolo et al., 2002; Ricart, 2011;)	2‐3 (Ciravolo et al., 2002)	No (Ricart, 2011)	ABCB1, ABCC1 (Linenberger, 2005; Linenberger et al., 2001; Takeshita et al., 2009;)	Yes (Takeshita, 2013)	Yes (Szczepanski et al., 2011)
Brentuximab vedotin	CD30	1) Hodgkin's lymphoma, systemic anaplastic large cell lymphoma (2011) (Younes et al., 2010) 2) Primary cutaneous anaplastic large cell lymphoma, CD30+ mycosis fungoides (2017) (Prince et al., 2017) 3) Peripheral T‐cell lymphomas (2018) (Horwitz et al., 2019)	1) Recombinant chimeric anti‐CD30 2) Protease‐cleavable valine‐citrulline peptide 3) MMAE (Blood, 2014)	4 (Blood, 2014)	Yes (Blood, 2014)	ABCB1 (Chen et al., 2020)	Yes (Chen et al., 2020)	Yes (Hansen et al., 2016)
Inotuzumab ozogamicin	CD22	B‐cell acute lymphoblastic leukaemia (2017) (Kantarjian et al., 2016)	1) Humanized anti‐CD22 mAb 2) Acid‐labile butanoic acid 3) Calicheamicin (Dijoseph et al., 2004)	6 (Garrett et al., 2019)	No (Ricart, 2011)	ABCB1 (Takeshita et al., 2009)	Yes (Paul et al., 2019)	Yes (Ayre et al., 2017)
Polatuzumab vedotin	CD79b	Diffuse large B‐cell lymphoma (2019) (Sehn et al., 2020)	1) Humanized anti‐CD79b 2) Protease‐cleavable valine‐citrulline peptide 3) MMAE (Dornan et al., 2009)	3.5 (European Medicines Agency, 2019)	Not reported	ABCB1 (Yu et al., 2015)	Not reported	Yes (Buschow et al., 2010)
Belantamab mafodotin	BCMA	Relapsed or refractory multiple myeloma (2020) (Lonial et al., 2020)	1) Afucosylated, humanized anti‐BCMA 2) Protease‐resistant maleimidocaproyl 3) MMAF (Lonial et al., 2020)	Not reported	Yes (Tai et al., 2014)	Not reported***	Yes (Lonial et al., 2020)	Yes (Perez‐Amill et al., 2020)
ADCs for solid tumors								
Trastuzumab emtansine	HER2	1) HER2+ advanced breast cancer (2013) (Hurvitz et al., 2013; Modi et al., 2020 Verma et al., 2012) 2) HER2+ early breast cancer (2019) (Von Minckwitz et al., 2019)	1) Humanized anti‐HER2 (trastuzumab) 2) Non‐reducible thioether 3) DM1 (Lewis Phillips et al., 2008)	3.5 (Lewis Phillips et al., 2008)	Yes (Barok et al., 2011; Junttila et al., 2011)	ABCB1, ABCC1, ABCC2, ABCG2, ABCC4 (Barok et al., 2020; Hunter et al., 2020; Le Joncour et al., 2019;Li et al., 2018, 2014)	Yes (Barok et al., 2014)	Yes (Andre et al., 2002; Barok et al., 2018; Le Joncour et al., 2019;)
Trastuzumab deruxtecan	HER2	HER2+ advanced breast cancer (2019) (Modi et al., 2020)	1) Humanized anti‐HER2 (trastuzumab) 2) Enzymatically cleavable glycine‐glycine‐phenylalanine‐glycine tetrapeptide‐based 3) Deruxtecan (Ogitani et al., 2016)	8 (Ogitani et al., 2016)	Yes (Ogitani et al., 2016)	Not reported****	Yes (Modi et al., 2020)	Yes (Andre et al., 2002; Barok et al., 2018; Le Joncour et al., 2019)
Enfortumab vedotin	Nectin‐4	Metastatic urothelial cancer (2019) (Rosenberg et al., 2019, 2020)	1) Fully human anti‐Nectin‐4 2) Protease‐cleavable valine‐citrulline peptide 3) MMAE (Challita‐Eid et al., 2016)	4 (Challita‐Eid et al., 2016)	No (Challita‐Eid et al., 2016)	Not reported***	Yes (Rosenberg et al., 2019)	Not reported
Sacituzumab govitecan	Trop‐2	Advanced triple negative breast cancer (2020) (Bardia et al., 2017, 2019)	1) Humanized anti‐Trop‐2 2) CL2A: pH‐sensitive, cleavable, PEG‐, maleimidocaproyl‐ and p‐aminobenzyloxycarbonyl‐containing 3) SN‐38 (Cardillo et al., 2011)	7.6 (Sharkey et al., 2015)	Yes (Varughese et al., 2011)	ABCG2 (Chang et al., 2016)	Yes (Bardia et al., 2017, 2019)	Yes (Trerotola et al., 2015)

Note: ABC drug efflux transporters are major contributors to multidrug resistance (MDR). ABCB1, ABCC1, ABCG2 and ABCA3 ABC drug efflux transporters have been described as EV‐related contributors to resistance during cancer chemotherapy (Bebawy et al., 2009; Corcoran et al., 2012; Maacha et al., 2019; Torreggiani et al., 2016; Zhang et al., 2014; ). ABCC2 and ABCC5 are also found in the EVs (Andrade et al., 2019).

Abbreviations: ATP, adenosine triphosphate; BCMA, B‐cell maturation antigen; DM1, Derivative of maytansine 1; HER2, Human epidermal growth factor receptor‐2; FDA, U.S. Food and Drug Administration; MMAE, Monomethyl auristatin E; SN‐38, 7‐ethyl‐10‐hydroxycamptothecin, an active metabolite of irinotecan; Trop‐2, Trophoblast cell‐surface antigen 2.

* Gemtuzumab ozogamicin became the first approved ADC in 2000 when the FDA approved it for the treatment of patients with CD33‐positive adult myeloid leukaemia (AML) who were not candidates for aggressive chemotherapy (Appelbaum & Bernstein, 2017). However, a post approval study in patients with de novo AML showed no overall improvement in survival and an increased treatment‐related mortality. These results led to the withdrawal of gemtuzumab ozogamicin from the market in 2010 (Appelbaum & Bernstein, 2017). In 2017, after evaluating new data on the clinical efficacy and safety of gemtuzumab ozogamicin administered on a fractionated dosing schedule, the FDA approved gemtuzumab ozogamicin for the treatment of newly‐diagnosed CD33‐positive AML in adults (Jen et al., 2018), and for the treatment of relapsed or refractory CD33‐positive AML in adults and in pediatric patients 2 years or older (Norsworthy et al., 2018).

** In 2020, this indication of gemtuzumab ozogamicin was extended for newly diagnosed CD33‐positive AML to include pediatric patients 1 month or older (Gamis et al., 2014).

*** To date, no ABC transporter‐related resistance has been reported with the recently approved belantamab mafodotin and enfortumab vedotin. They have auristatin payloads, MMAF and MMAE, respectively. Importantly, the membrane permeable MMAE‐type payloads are known substrates of the ABC drug efflux transporters, while the less permeable MMAF‐type payloads are susceptible to efflux pumps (Moquist et al., 2020).

**** Trastuzumab deruxtecan is effective for cancers that have become resistant to trastuzumab emtansine due to overexpression of ABC drug efflux transporters (Takegawa et al., 2017).

### 2.1 ADC structure

**FIGURE 1:**
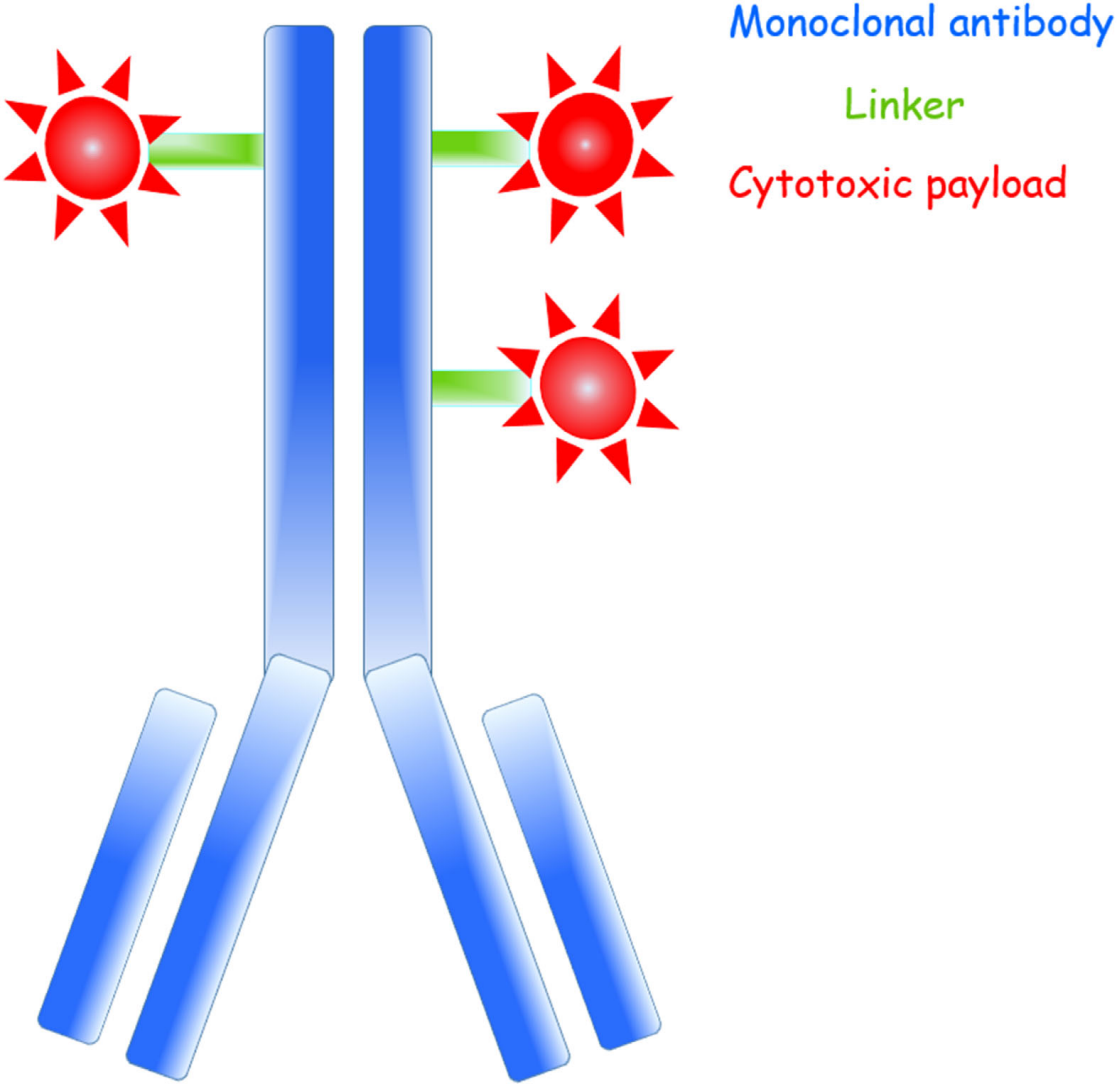
A schematic structure of an antibody‐drug conjugate (ADC) with a drug‐antibody ratio (DAR) of three.

An ADC consists of a monoclonal antibody (mAb), a cytotoxic payload, and a linker joining the two (Figure 1). The mAb typically targets an antigen that is highly expressed on the cancer cell plasma membrane and whose expression is low on non‐malignant cells. Using a mAb as the carrier for the toxic payload allows reaching high selectivity, stability, and often a favourable pharmacokinetic profile for the ADC (Beck et al., 2017; Khongorzul et al., 2020). The cytotoxic payloads are covalently bound to the mAb through a linker. The linker should prevent the release of the cytotoxic drug into the circulation or in the off‐target tissues (Beck et al., 2017; Khongorzul et al., 2020), and it should ideally allow the release of the payloads when the ADC has first been internalized into the target cancer cell (Barok et al., 2014; Khongorzul et al., 2020). The payloads are usually highly cytotoxic small‐molecule drugs (Beck et al., 2017; Khongorzul et al., 2020).

The cytotoxic drug‐antibody ratio (DAR) varies between the ADCs. In general, a high DAR might result in the delivery of more payloads into the cancer cells, and, therefore, could increase the ADC potency. However, a high DAR may also lead to a less favourable pharmacokinetic profile, and, therefore, a low DAR may sometimes be preferable (Beck et al., 2017; Barfield et al., 2020; Khongorzul et al., 2020). The DAR of the approved nine ADCs ranges from 2 and 8 (Table 1). The linkage of the payloads to the mAb should preferably not affect the binding affinity of the mAb to its molecular target or inhibit the potential inherent anti‐tumour effects of the mAb. For successful ADC therapy the target protein needs to be well selected, and all ADC components, the antibody, the linker, and the payload and the DAR need to be carefully optimized.

### 2.2 Mechanism of action of the ADCs

**FIGURE 2:**
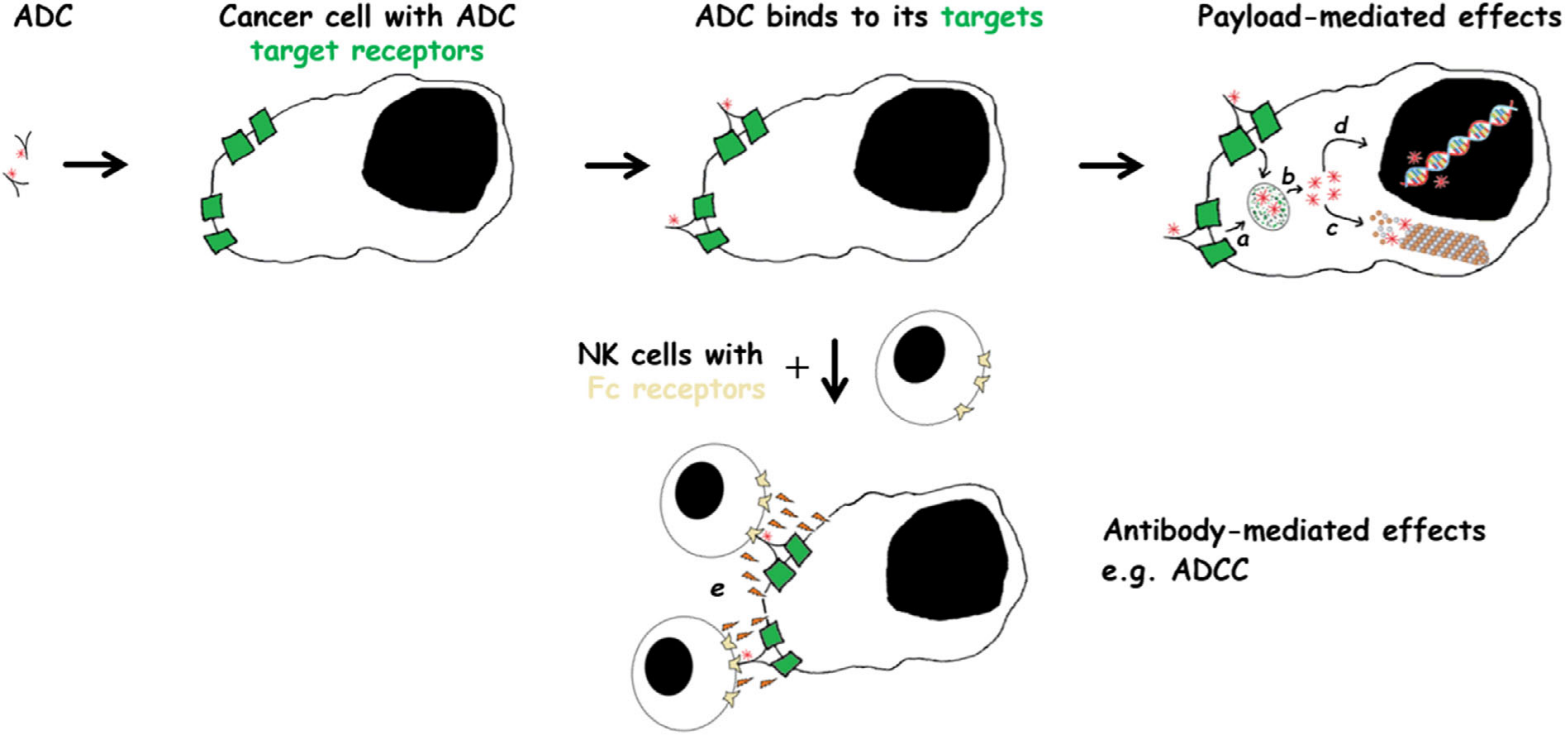
The mechanism of action of antibody‐drug conjugates (ADCs). [ADCs may cause target cancer cell death via releasing the cytotoxic payload within the target cell. Following internalization (a), the cytotoxic payloads are released after lysosomal cleavage or enzymatic degradation (b). The intracellularly released cytotoxic payloads inhibit cellular functions, e.g., tubulin polymerization (c), or may cause DNA damage (d). The targeting antibody may also have anti‐cancer effects. For example, it may elicit the host immune response that may involve several types of immune cells, such as natural killer (NK) cells. The Fc part of the ADC binds to the Fc receptor bearing NK cells leading to the release of cytotoxic perforin and granzyme from the NK cells (e). Abbreviation: ADCC, antibody‐dependent cellular cytotoxicity].

After intravenous administration, the ADC delivers the cytotoxic payloads into the cells that express the antibody target. Once bound to the target, the cells internalize the ADC‐receptor complex via receptor‐mediated endocytosis. The cytotoxic payloads are released within the target cell once the ADC has been cleaved in the acidic environment of the lysosomes or by enzymatic degradation in the lysosomes. The intracellularly released cytotoxic payloads and their metabolites interfere with the cellular machinery causing cell death (Barok et al., 2014; Khongorzul et al., 2020) (Figure 2). To date, the payloads that are used in ADCs cause cell death by inhibiting tubulin polymerization (e.g., monomethyl auristatins, and maytansines), cause direct DNA damage (e.g., calicheamicin), or inhibit topoisomerases (e.g., deruxtecan and SN‐38) (Supplementary Table 1). Some payloads used in ADCs have an undisclosed mechanism of action (Beck et al., 2017; Khongorzul et al., 2020).

Five out of the nine approved ADCs have a mAb that has shown anti‐tumour effects on its own even without the payload (brentuximab vedotin, trastuzumab emtansine, trastuzumab deruxtecan, sacituzumab govitecan, and belantamab mafodotin (Ansell, 2014; Barok et al., 2007, 2011; Junttila et al., 2011; Ogitani et al., 2016; Tai et al., 2014; Varughese et al., 2011) (Table 1). Consequently, these ADCs may have a dual mechanism of action on cancer consisting of the anti‐tumour effects related to the plain mAb and those associated with the intracellular release of the cytotoxic payload and its metabolites within the target cells (Barok et al., 2014) (Figure 2).

### 2.3 Resistance to ADCs

Emergence of drug resistance has been reported with almost all of the approved ADCs including gemtuzumab ozogamicin, brentuximab vedotin, and trastuzumab emtansine (Table 1) (Barok et al., 2014; Chen et al., 2020; Takeshita, 2013). Thus, resistance to ADCs is frequent and concerns a wide variety of cancers.

There are several mechanisms that can cause resistance to ADCs. These include *i*) impaired binding of the ADC to the target antigens, *ii*) attenuated ADC internalization into cancer cells, *iii*) defects in ADC intracellular trafficking, *iv*) impaired lysosomal function in ADC degradation, *v*) changes in the cell cycle dynamics (e.g., altered expression of cell‐cycle proteins that control G_2_‐M transition, or cell cycle arrest with cell dormancy), *vi*) activation of cell survival pathways, and *vii*) drug efflux pumps (Barok et al., 2014; Collins et al., 2019; García‐Alonso et al., 2018).

Drug efflux pumps appear to be a prominent resistance mechanism to ADCs. Cytotoxic drugs can be disposed by drug efflux pumps from the cytoplasm of cancer cells by the ATP binding cassette (ABC) transporters potentially resulting in clinical failure of chemotherapy (Yu et al., 2013). Auristatins, maytansines, calicheamicin and irinotecans, which are commonly used cytotoxic payloads in the ADCs, are all substrates for the multidrug resistant drug efflux transporters (Dan et al., 2018; Parslow et al., 2016; Xu & Villalona‐Calero, 2002). Of note, the ABC transporters contribute to the resistance of at least six out of the nine approved ADCs (Chang et al., 2016; Chen et al., 2020; Le Joncour et al., 2019; Li et al., 2018; Linenberger et al., 2001, 2005; Ricart, 2011; Takeshita et al., 2009; Yu et al., 2015) (Table 1).

## 3 EXTRACELLULAR VESICLES

Besides the mechanisms discussed above, EVs may contribute to the efficacy and safety of ADCs. EVs contain proteins, lipids, various RNA species, DNA, and metabolites. EVs can transfer their cargo not only into cancer cells and tumour stromal cells, but also to distant non‐malignant cells, where their contents remain active, and influence the biological functions of the recipient cells (Al‐Nedawi et al., 2009; Puhka et al., 2017; Ratajczak et al., 2006; Valadi et al., 2007; Van Niel et al., 2018). Consequently, EVs have an important role in the cell‐cell communication.

EVs include exosomes (up to 150 nm in diameter; generated by inward budding of endosomes that mature to multivesicular bodies), microvesicles (with size up to 1000 nm; directly shed from the plasma membrane of the cells), apoptotic bodies (1 to 2 μm in diameter), and other types of membrane vesicles with varying sizes, functional properties, and biogenesis (Becker et al., 2016; Van Niel et al., 2018). All types of cells, including cancer cells, appear to secrete EVs (Becker et al., 2016; Van Niel et al., 2018).

EVs can be taken up by the recipient cells with several mechanisms. These include *i*) receptor‐mediated endocytosis, *ii*) fusion with the target cell membrane, *iii*) phagocytosis, and *iv*) macropinocytosis (Mulcahy et al., 2014; Van Niel et al., 2018). Following the uptake, the EV‐delivered cargo can enter the cytoplasm and/or the nucleus of the recipient cell, and alter cell biological functions (Pitt et al., 2016; Van Niel et al., 2018). Alternatively, the EV‐delivered cargo can be directed to the lysosomes and degraded (Jakhar & Crasta, 2019; Tian et al., 2013; Van Niel et al., 2018).

Cancer cells secrete significant amounts of EVs (Becker et al., 2016) that can be isolated from the body fluids such as the blood, the saliva, and the urine (Becker et al., 2016; Boukouris & Mathivanan, 2015). The quantity and quality of these EVs may change upon drug treatments and the development of drug resistance (Namee & O'driscoll, 2018). EVs are pivotal mediators of cell‐cell communication between cancer cells, and also between cancer cells and the stromal cells both within the cancer microenvironment and at distant tissues (Becker et al., 2016; Kalluri, 2016; Kosaka et al., 2016). EVs have been implicated in the modulation of cancer growth and metastasis, tumour angiogenesis, and anti‐cancer immunity (Becker et al., 2016; Kalluri, 2016; Kosaka et al., 2016). EVs can also alter the effects of anti‐cancer drugs in multiple ways, sometimes leading to drug resistance (Becker et al., 2016; Maacha et al., 2019; Namee & O'driscoll, 2018). We discuss below how EVs can influence the effects of the two major components of ADCs, the small molecule anti‐cancer drugs and the mAbs.

### 3.1 Resistance to small molecule anti‐cancer drugs through EVs

Elimination of small molecule anti‐cancer drugs from the cancer cells contributes to systemic cancer therapy resistance (Yu et al., 2013). Several types of cancer cells may utilize EVs as an efflux mechanism of small molecule anti‐cancer drugs at least in two ways. First, cancer cells can accumulate small molecule anti‐cancer drugs into the EVs, and then dispose the drug‐loaded EVs into the extracellular space (Federici et al., 2014; Koch et al., 2016; Maacha et al., 2019; Safaei et al., 2005; Shedden et al., 2003). Second, chemotherapy‐resistant cancer cells may secrete EVs loaded with ABC drug efflux transporters, which are major contributors to the multidrug resistance in cancer (W. Robey et al., 2011; Yu et al., 2013), and, subsequently, chemotherapy‐sensitive cancer cells may take up such efflux‐transporter containing EVs from the extracellular space and receive functional efflux pump proteins, turning drug‐sensitive cancer cells into resistant ones (Bebawy et al., 2009; Corcoran et al., 2012; Maacha et al., 2019; Torreggiani et al., 2016; Zhang et al., 2014).

The microRNA cargo of the EVs may also promote resistance to small molecule anti‐cancer drugs via regulation of gene expression (Becker et al., 2016; Maacha et al., 2019). EVs may deliver also a variety of other factors, such as PDGFR‐β, hepatocyte growth factor, TGF‐β, or intermediary metabolites, which may support the survival pathways of the recipient cancer cells (Maacha et al., 2019; Zhao et al., 2016).

### 3.2 EV‐mediated resistance to anti‐cancer mAb therapy

Rituximab is an anti‐CD20 mAb approved for the treatment of patients with CD20‐positive non‐Hodgkin's lymphoma or chronic lymphocytic leukaemia (Hiddemann et al., 2005; Nabhan & Rosen, 2014). Complement‐dependent cytotoxicity has a pivotal role in the anti‐cancer efficacy of rituximab (Smith, 2003). B‐cell lymphoma cells release EVs that carry CD20, and rituximab binds to such EVs. The binding of rituximab leads to the fixation of complement on the surface of the EVs. This decoy effect, mediated by the CD20+ EVs, results in the consumption of both complement and free rituximab leading to impaired efficacy of rituximab on the target cancer cells (Aung et al., 2011; Oksvold et al., 2014).

Trastuzumab, an anti‐HER2 mAb drug approved for the treatment of patients with HER2‐positive breast cancer and gastric cancer (Bang et al., 2010; Slamon et al., 2001), has a direct inhibitory effect on HER2‐positive cancer cells (Barok et al., 2011; Köninki et al., 2010). Trastuzumab also recruits immune effector cells that kill the cancer cells via antibody‐dependent cellular cytotoxicity (ADCC) (Barok et al., 2007, 2008). HER2‐positive cancer cells secrete EVs that carry HER2, and trastuzumab binds to such EVs (Andre et al., 2002; Battke et al., 2011; Ciravolo et al., 2012). The HER2+ EV‐mediated decoy effect may lead to the attenuation of the direct cancer growth inhibitory effect of trastuzumab and the ADCC‐related cytotoxic effect of trastuzumab (Battke et al., 2011; Ciravolo et al., 2012).

Taken together, EVs are a means by which cancer cells may gain resistance to both small molecule anti‐cancer drugs and anti‐cancer mAbs, the two key components of the ADCs.

## 4 INFLUENCE OF EVs ON THE EFFICACY OF ADCs

Approximately 100 ADCs are being investigated in clinical trials (Chau et al., 2019; Coats et al., 2019; Khongorzul et al., 2020). More than 50 known antigens expressed on cancer cells have been selected as ADC targets (Khongorzul et al., 2020), and at least 26 of these ADC target‐antigens are present also on cancer‐derived EVs (Supplementary Table 1). EVs express the target antigen of at least 69 ADCs, of which 43 are currently in clinical trials or have been approved, two are in the preclinical phase, and the clinical development of 24 has been discontinued (Supplementary Table 1). Of note, the target antigen of at least eight out of the nine approved anti‐cancer ADCs is expressed also on EVs (Table 1, Supplementary Table 1). After binding to their targets on the EVs, the EVs deliver the ADCs to the EV recipient cells (Barok et al., 2018; Hansen et al., 2016). Since EVs can deliver their cargo to both local and distant cancerous and non‐cancerous cells, EVs may influence the efficacy of the ADCs.

### 4.1 EVs and the bystander effect

**FIGURE 3:**
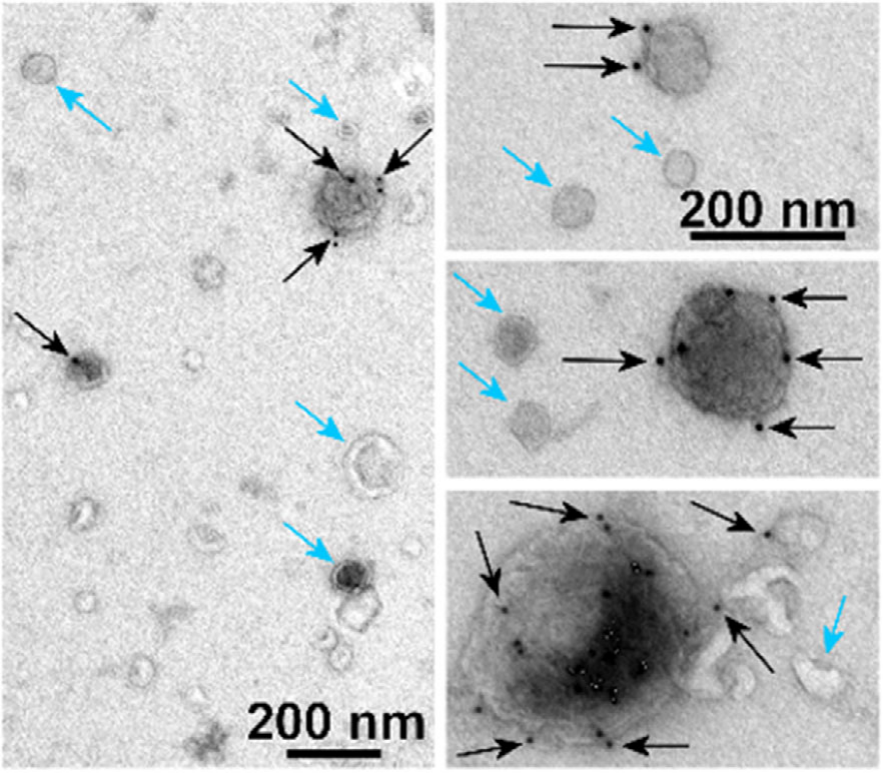
Cancer cells secrete extracellular vesicles (EVs) that may carry antibody‐drug conjugates (ADCs). [Extracellular vesicles isolated from the cell culture medium of human HER2+ gastric cancer cells (RN87) resistant to an anti‐HER2 ADC trastuzumab emtansine. The cells were cultured in the presence of trastuzumab emtansine. EVs were whole‐mount immuno‐stained with 12 nm gold‐conjugated anti‐human antibody that recognizes trastuzumab emtansine on the EV surface (black dots). The electron microscopy images show EVs of different sizes including vesicles carrying trastuzumab emtansine (black dots and arrows) and vesicles without trastuzumab emtansine (blue arrows). Bar = 200 nm].

**FIGURE 4:**
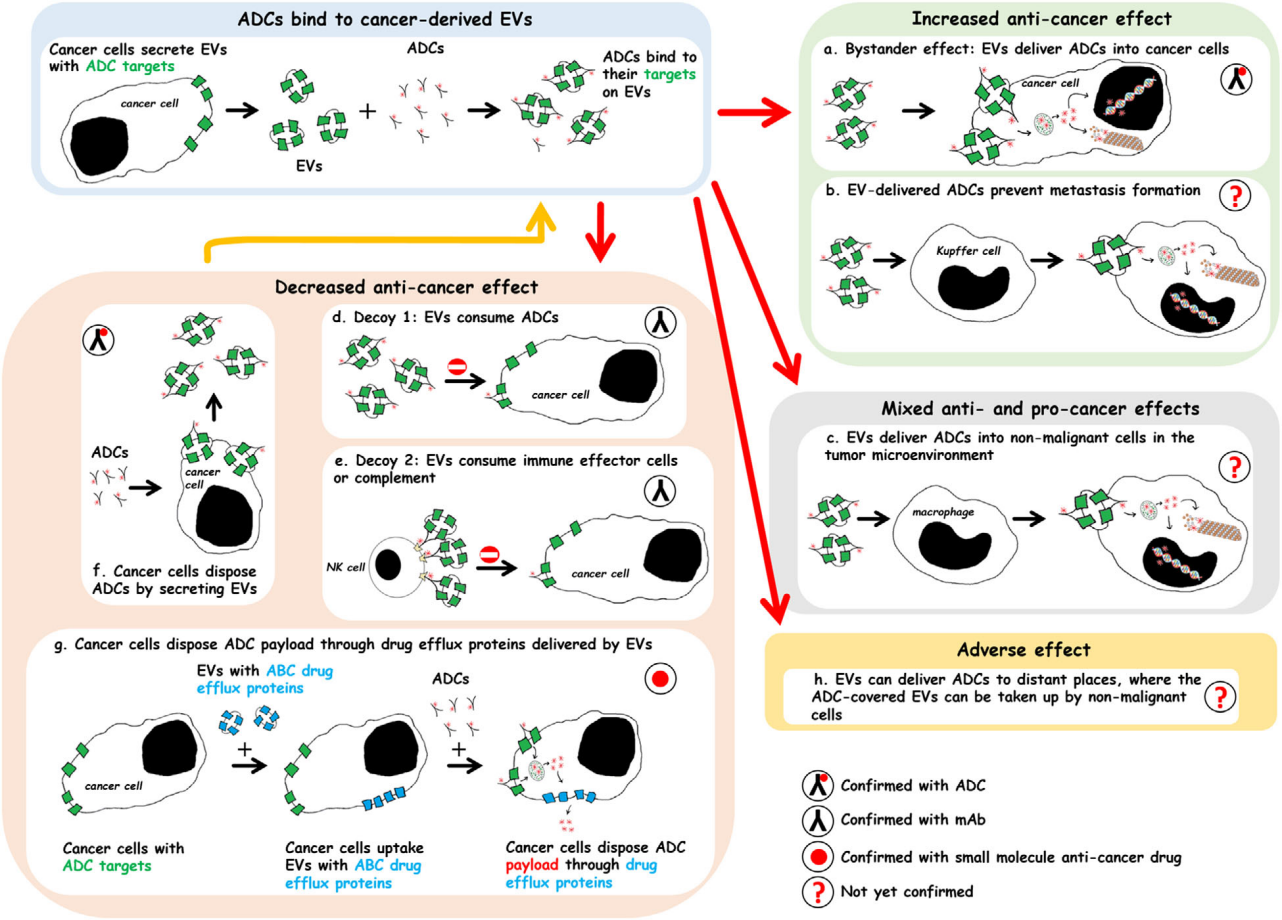
Extracellular vesicles (EVs) can alter the efficacy of antibody‐drug conjugates (ADCs). [ADCs bind to EVs secreted by cancer cells that express the ADC target proteins. Such EVs can deliver ADCs to both local and distant cancerous and non‐cancerous cells leading to anti‐cancer and pro‐cancer effects, and could modify the ADC‐related adverse effects. EVs can also contribute to the resistance of cancer to ADCs. As a bystander effect, EV‐delivered ADCs may cause the death of the neighbouring tumor cells (a). EV‐delivered ADCs can inhibit Kupffer cells in the liver responsible for pre‐metastatic niche formation (b). EVs can also deliver ADCs into non‐malignant cells in the tumor microenvironment that may lead to either cancer growth promoting or inhibiting effects depending on the role of the recipient cells in cancer progression (c). EVs can cause ADC‐resistance through EV‐mediated decoy mechanisms (d and e), expulsion of ADCs into the extracellular space by EV secretion (this can contribute to the pool of extracellular EV‐ADCs; an orange arrow) (f), or EVs can transfer ABC drug efflux transporters into ADC‐sensitive cancer cells where the transporters may pump out the ADC payloads from the cells (g). EVs can carry ADCs into distant non‐malignant cells contributing to ADC toxicity (h). The circled labels indicate whether the mechanism has been observed with ADCs, monoclonal antibodies, small molecule anti‐cancer drugs, or whether it is still hypothetical. Abbreviations: ADC, antibody‐drug conjugate; EV, extracellular vesicle; mAbs, monoclonal antibody].

The anti‐CD30 ADC brentuximab vedotin binds to CD30‐positive EVs released from lymphoma cells. CD30‐negative lymphoma cells may take up brentuximab vedotin‐covered EVs leading to their apoptotic death (Hansen et al., 2016). Similarly, the anti‐HER2 ADC, trastuzumab emtansine, has its target on the EVs derived from HER2‐positive cancer cells (Barok et al., 2018; Andre et al., 2002). Trastuzumab emtansine binds to these EVs (Figure 3), and may be carried to other cancer cells via the EVs leading to apoptotic death of the recipient cancer cells (Barok et al., 2018). These observations suggest that EVs can deliver ADCs into the neighbouring cancer cells that lack the antibody target protein leading to a bystander effect, which may increase the anti‐cancer efficacy of the ADCs (Figure 4).

An EV‐mediated bystander effect may be of importance also with other ADC targets. For example, glioma cells secrete EVs that carry the epidermal growth factor receptor variant III (EGFRvIII), and prostate cancer cells secrete EVs that express trophoblast cell‐surface antigen 2 (Trop‐2), and such EVs may deliver their cargo into cancer cells within the same tumour that lack EGFRvIII or Trop‐2 expression (Al‐Nedawi et al., 2008; Trerotola et al., 2015). At present, one anti‐EGFRvIII ADC (ABT‐414) is being evaluated in clinical trials (Goss et al., 2018), whereas the clinical development of another anti‐EGFRvIII ADC was discontinued (Hamblett et al., 2015) (Supplementary Table 1). Sacituzumab govitecan is an approved anti‐Trop‐2 ADC (Bardia et al., 2019) (Table 1), while the clinical development of PF‐06664178, another anti‐Trop‐2 ADC, was stopped (King et al., 2018) (Supplementary Table 1). Hypothetically, EVs might deliver anti‐EGFRvIII or anti‐Trop‐2 ADCs into cancer cells that lack EGFRvIII or Trop‐2 expression. This might increase the anti‐cancer effect of these ADCs.

### 4.2 EV delivery to tumour stromal cells

Besides cancer cells, non‐malignant cells may take up cancer‐derived EVs. For example, endothelial cells accumulated tissue factor‐containing EVs derived from mesenchymal‐like cancer cells (Garnier et al., 2012) and epidermal growth factor receptor (EGFR)‐containing EVs originating from human squamous carcinoma cells (Al‐Nedawi et al., 2009). Similarly, progenitor smooth muscle cells took up KIT‐containing EVs derived from gastrointestinal stromal tumour cells (Atay et al., 2014), and monocytes integrin αvβ6‐containing EVs originating from prostate cancer cells (Lu et al., 2018). These observations indicate that cancer‐derived EVs can transfer their contents into tumour stromal cells (Peinado et al., 2017). Tisotumab vedotin, an ADC targeting tissue factor (De Bono et al., 2019), and several ADCs against EGFR are currently being investigated in clinical trials, while the clinical development of an anti‐KIT ADC and an anti‐integrin αv ADC (LOP628 (Abrams et al., 2018) and IMGN388 (Bendell et al., 2010; Raab‐Westphal et al., 2017), respectively) has been discontinued (Supplementary Table 1).

EVs that express the ADC target may thus be able to deliver ADCs into non‐malignant cells in the tumour microenvironment, which could have either cancer growth promoting or inhibiting effects depending on the role of the recipient cells in cancer progression. For example, EV‐mediated inhibition of tumour growth promoting intratumoral macrophages, fibroblasts, or endothelial cells might inhibit cancer growth, whereas damaging of intratumoral anti‐cancer immune cells could enhance tumour growth (Figure 4).

### 4.3 EVs and resistance to ADCs

Similar resistance mechanisms may operate with the ADCs as for the corresponding plain antibodies and small molecule anti‐cancer drugs. Trastuzumab is the mAb part of the two approved anti‐HER2 ADCs (trastuzumab emtansine and trastuzumab deruxtecan (Barok et al., 2014; Ogitani et al., 2016)), and it is the mAb component of six other anti‐HER2 ADCs that are being evaluated in clinical trials (ADCT‐502, ALT‐P7, A166, BAT8001, PF‐06804103, and trastuzumab duocarmazine) (Supplementary Table 1 and 2). The efficacy of the trastuzumab‐based anti‐HER2 ADCs and other ADCs might be altered by EV‐mediated decoy mechanisms (Figure 4). HER2‐positive gastric cancer cells resistant to trastuzumab emtansine discarded trastuzumab emtansine into the extracellular space by EV secretion (Le Joncour et al., 2019), and since a sufficient intracellular concentration of the cytotoxic payload DM1 is essential for the anti‐cancer activity (Barok et al., 2014), expulsion of DM1 may contribute to trastuzumab emtansine resistance (Figure 4). The cytotoxic payloads commonly used in ADCs are substrates of the ABC drug efflux transporters (Dan et al., 2018; Parslow et al., 2016), and, the presence of ABC drug efflux transporters on cancer cells decreases ADC efficacy (Barok et al., 2020; Chen et al., 2020; Hunter et al., 2020; Li et al., 2018; Le Joncour et al., 2019; Linenberger, 2005; Linenberger et al., 2001; Takeshita et al., 2009) (Table 1). Therefore, EVs may attenuate the anti‐cancer efficacy of ADCs by transferring ABC drug efflux transporters into drug‐sensitive cancer cells contributing to treatment resistance (Figure 4).

### 4.4 EV‐delivered ADCs in prevention of metastasis

EVs have been implicated in pre‐metastatic niche formation (Wortzel et al., 2019). For example, EVs derived from gastric cancer cells can transfer human epidermal growth factor receptors (EGFR) into liver stromal cells, which leads to changes in the regulation of the liver microenvironment and may facilitate the formation and growth of gastric cancer liver metastases (Zhang et al., 2017). Similarly, cancer‐derived EVs delivering integrin αvβ5 specifically bind to the Kupffer cells in the liver, and facilitate pre‐metastatic niche formation and liver metastases from breast cancer and pancreatic cancer (Hoshino et al., 2015). Hypothetically, delivering of anti‐EGFR or anti‐integrin αv ADCs using EVs into the liver might attenuate the EV‐mediated metastasis formation (Figure 4). Four anti‐EGFR ADCs are currently studied in clinical trials (ABBV‐321, ABT‐414, AVID‐100, and MRG003 (Goss et al., 2018; O'Connor‐McCourt et al., 2016; Xu et al., 2020)), whereas the clinical development of another anti‐EGFR ADC (IMGN289) (Setiady et al., 2014) and an anti‐integrin αv ADC (IMGN388 (Bendell et al., 2010; Raab‐Westphal et al., 2017) were discontinued (Supplementary Table 1).

## 5 EVs AND ADC SAFETY

Administration of ADCs is often associated with off‐target adverse effects. These are mainly driven by the payload, and may be promoted by the mAb and the linker (Birrer et al., 2019; Donaghy, 2016; Wolska‐Washer & Robak, 2019). ADCs can induce toxicity through their low affinity binding to the target antigen, or non‐specific binding to the Fc receptors. Early release of the payload in the circulation or in off‐target tissues due to linker instability results in increased systemic exposure and adverse effects (Birrer et al., 2019; Donaghy, 2016; Wolska‐Washer & Robak, 2019). This may lead to stopping of the treatment (Wolska‐Washer & Robak, 2019), and even to discontinuation the clinical development program of an ADC due to a poor therapeutic index (Coats et al., 2019).

EVs could influence also the side effects of anti‐cancer ADCs, but this remains a hypothesis in the absence of research data. Cancer‐derived EVs found in the blood and in other body fluids (Al‐Nedawi et al., 2008; Becker et al., 2016; Boukouris & Mathivanan, 2015; Ciardiello et al., 2016; Hoshino et al., 2015; Melo et al., 2015) may transfer their contents besides cancer to the recipient cells in non‐cancerous tissues (Peinado et al., 2017), which could lead to side effects. For example, cancer‐derived EVs deliver EGFR and integrin αv into liver stromal cells as discussed above (Hoshino et al., 2015; Zhang et al., 2017), which could contribute to liver toxicity of anti‐EGFR ADCs and anti‐integrin αv ADCs (Figure 4).

## 6 CONCLUSIONS

ADCs are one of the fastest‐growing class of anti‐cancer drugs with nine members currently approved by the FDA, and approximately 100 members in the clinical development (Chau et al., 2019; Coats et al., 2019; Khongorzul et al., 2020). At least 26 ADC target‐antigens are present on cancer‐derived EVs, and there are at least 69 ADCs, including eight out of the nine approved anti‐cancer ADCs, whose target‐antigens are expressed on the EVs. Since EVs can transfer their content not only into cancer cells and tumour stromal cells, but also into distant non‐malignant cells, they have a potential to mediate both the anti‐cancer effects and the adverse effects of the ADCs. The EVs may contribute to the success and failure of ADC therapy with several mechanisms, but some of these mechanisms remain still hypothetical and require confirmation.

## CONFLICTS OF INTEREST

HJ is the Chair of the Scientific Advisory Board at Orion Pharma and at Neutron Therapeutics Ltd. The other authors do not declare conflicts of interest.

## FUNDING

Academy of Finland, Cancer Society of Finland, Sigrid Juselius Foundation, Jane and Aatos Erkko Foundation, Helsinki University Research Grants.

## SEARCH STRATEGY AND REFERENCE SELECTION CRITERIA

To find all relevant studies, we searched the PubMed, the ClinicalTrials.gov website, Vesiclepedia, and ExoCarta. Other literature sources were also searched (Google, ADC Review). We also searched abstracts from the annual meetings of the American Association for Cancer Research, and the American Society for Clinical Oncology. Various combinations of search terms were used depending on the requirements of the database being searched. These terms included: ‘antibody‐drug conjugate’, ‘ADC’, ‘ATP binding cassette transporter’, ‘ABC transporter’, ‘cancer’, ‘drug efflux pump’, ‘exosome’, ‘extracellular vesicle’, ‘EV’, ‘failure of chemotherapy’, ‘resistance’, and the names of all individual ADCs mentioned in this paper or found in relevant papers. We searched for the ADC targets in the extracellular vesicle databases (Vesiclepedia, ExoCarta). We also searched the references listed in relevant papers.

## Supporting information

Supplementary Table 1: Antibody‐drug conjugates (ADC) and their targets expressed on extracellular vesicles (EVs)

Supplementary Table 2: Trastuzumab‐based anti‐HER2 antibody‐drug conjugates

## References

[jev212070-bib-0001] Abrams, T. , Connor, A. , Fanton, C. , Cohen, S. B. , Huber, T. , Miller, K. , Hong, E. E. , Niu, X. , Kline, J. , Ison‐Dugenny, M. , Harris, S. , Walker, D. , Krauser, K. , Galimi, F. , Wang, Z. , Ghoddusi, M. , Mansfield, K. , Lee‐Hoeflich, S. T. , Holash, J. , … Schleyer, S. C. (2018). Preclinical antitumor activity of a novel anti‐c‐KIT antibody‐drug conjugate against mutant and wild‐type c‐KIT‐positive solid tumors. Clinical Cancer Research, 24(17), 4297–4308. 29764854 10.1158/1078-0432.CCR-17-3795

[jev212070-bib-0002] Al‐Nedawi, K. , Meehan, B. , Kerbel, R. S. , Allison, A. C. , & Rak, J. (2009). Endothelial expression of autocrine VEGF upon the uptake of tumor‐derived microvesicles containing oncogenic EGFR. PNAS, 106(10), 3794–3799. 19234131 10.1073/pnas.0804543106 PMC2656159

[jev212070-bib-0003] Al‐Nedawi, K. , Meehan, B. , Micallef, J. , Lhotak, V. , May, L. , Guha, A. , & Rak, J. (2008). Intercellular transfer of the oncogenic receptor EGFRvIII by microvesicles derived from tumour cells. Nature Cell Biology, 10(5), 619–624. 18425114 10.1038/ncb1725

[jev212070-bib-0004] Al‐Nedawi, K. , Meehan, B. , & Rak, J. (2009). Microvesicles: Messengers and mediators of tumor progression. Cell Cycle (Georgetown, Tex), 8(13), 2014–2018. 10.4161/cc.8.13.8988 19535896

[jev212070-bib-0005] Andrade, L. N. S. , Otake, A. H. , Cardim, S. G. B. , da Silva, F. I. , Ikoma Sakamoto, M. M. , Furuya, T. K. , Uno, M. , Pasini, F. S. , & Chammas, R. (2019). Extracellular Vesicles Shedding Promotes Melanoma Growth in Response to Chemotherapy. Scientific Reports, 9(1), 14482. 31597943 10.1038/s41598-019-50848-z PMC6785560

[jev212070-bib-0006] Andre, F. , Schartz, N. E. , Movassagh, M. , Flament, C. , Pautier, P. , Morice, P. , Pomel, C. , Lhomme, C. , Escudier, B. , Le Chevalier, T. , Tursz, T. , Amigorena, S. , Raposo, G. , Angevin, E. & Zitvogel, L. (2002). Malignant effusions and immunogenic tumour‐derived exosomes. Lancet, 360(9329), 295–305. 12147373 10.1016/S0140-6736(02)09552-1

[jev212070-bib-0007] Ansell, S. M. (2014). Brentuximab vedotin. Blood, 124(22), 3197–3200. 25293772 10.1182/blood-2014-06-537514

[jev212070-bib-0008] Appelbaum, F. R. , & Bernstein, I. D. (2017). Gemtuzumab ozogamicin for acute myeloid leukemia. Blood, 130(22), 2373–2376. 29021230 10.1182/blood-2017-09-797712

[jev212070-bib-0009] Atay, S. , Banskota, S. , Crow, J. , Sethi, G. , Rink, L. , & Godwin, A. K. (2014). Oncogenic KIT‐containing exosomes increase gastrointestinal stromal tumor cell invasion. PNAS, 111(2), 711–716. 24379393 10.1073/pnas.1310501111 PMC3896203

[jev212070-bib-0010] Aung, T. , Chapuy, B. , Vogel, D. , Wenzel, D. , Oppermann, M. , Lahmann, M. , Weinhage, T. , Menck, K. , Hupfeld, T. , Koch, R. , Trumper, L. , & Wulf, G. G. (2011). Exosomal evasion of humoral immunotherapy in aggressive B‐cell lymphoma modulated by ATP‐binding cassette transporter A3. PNAS, 108(37), 15336–15341. 21873242 10.1073/pnas.1102855108 PMC3174603

[jev212070-bib-0011] Ayre, D. C. , Chute, I. C. , Joy, A. P. , Barnett, D. A. , Hogan, A. M. , Grull, M. P. , Pena‐Castillo, L. , Lang, A. S. , Lewis, S. M. , & Christian, S. L. (2017). CD24 induces changes to the surface receptors of B cell microvesicles with variable effects on their RNA and protein cargo. Scientific Reports, 7(1), 8642. 28819186 10.1038/s41598-017-08094-8 PMC5561059

[jev212070-bib-0012] Bang, Y.‐J. , Van Cutsem, E. , Feyereislova, A. , Chung, H. C. , Shen, L. , Sawaki, A. , Lordick, F. , Ohtsu, A. , Omuro, Y. , Satoh, T. , Aprile, G. , Kulikov, E. , Hill, J. , Lehle, M. , Rüschoff, J. , & Kang, Y.‐K. (2010). Trastuzumab in combination with chemotherapy versus chemotherapy alone for treatment of HER2‐positive advanced gastric or gastro‐oesophageal junction cancer (ToGA): A phase 3, open‐label, randomised controlled trial. Lancet, 376(9742), 687–697. 20728210 10.1016/S0140-6736(10)61121-X

[jev212070-bib-0013] Bardia, A. , Mayer, I. A. , Diamond, J. R. , Moroose, R. L. , Isakoff, S. J. , Starodub, A. N. , Shah, N. C. , O'shaughnessy, J. , Kalinsky, K. , Guarino, M. , Abramson, V. , Juric, D. , Tolaney, S. M. , Berlin, J. , Messersmith, W. A. , Ocean, A. J. , Wegener, W. A. , Maliakal, P. , Sharkey, R. M. , … Vahdat, L. T. (2017). Efficacy and safety of Anti‐Trop‐2 antibody drug conjugate sacituzumab govitecan (IMMU‐132) in heavily pretreated patients with metastatic triple‐negative breast cancer. Journal of Clinical Oncology, 35(19), 2141–2148. 28291390 10.1200/JCO.2016.70.8297 PMC5559902

[jev212070-bib-0014] Bardia, A. , Mayer, I. A. , Vahdat, L. T. , Tolaney, S. M. , Isakoff, S. J. , Diamond, J. R. , O'shaughnessy, J. , Moroose, R. L. , Santin, A. D. , Abramson, V. G. , Shah, N. C. , Rugo, H. S. , Goldenberg, D. M. , Sweidan, A. M. , Iannone, R. , Washkowitz, S. , Sharkey, R. M. , Wegener, W. A. , & Kalinsky, K. (2019). Sacituzumab Govitecan‐hziy in refractory metastatic triple‐negative breast cancer. New England Journal of Medicine, 380(8), 741–751. 10.1056/NEJMoa1814213 30786188

[jev212070-bib-0015] Barfield, R. M. , Kim, Y. C. , Chuprakov, S. , Zhang, F. , Bauzon, M. , Ogunkoya, A. O. , Yeo, D. , Hickle, C. , Pegram, M. D. , Rabuka, D. , & Drake, P. M. (2020). A Novel HER2‐targeted antibody‐drug conjugate offers the possibility of clinical dosing at trastuzumab‐equivalent exposure levels. Molecular Cancer Therapeutics, 19(9), 1866–1874. 32651200 10.1158/1535-7163.MCT-20-0190

[jev212070-bib-0016] Barok, M. , Balázs, M. , Nagy, P. , Rákosy, Z. , Treszl, A. , Tóth, E. , Juhász, I. , Park, J. W. , Isola, J. , Vereb, G. , & Szöllősi, J. (2008). Trastuzumab decreases the number of circulating and disseminated tumor cells despite trastuzumab resistance of the primary tumor. Cancer Letters, 260(1‐2), 198–208. 18096313 10.1016/j.canlet.2007.10.043

[jev212070-bib-0017] Barok, M. , Isola, J. , Pályi‐Krekk, Z. , Nagy, P. , Juhász, I. , Vereb, G. , Kauraniemi, P. , Kapanen, A. , Tanner, M. , Vereb, G. , & Szöllösi, J. (2007). Trastuzumab causes antibody‐dependent cellular cytotoxicity‐mediated growth inhibition of submacroscopic JIMT‐1 breast cancer xenografts despite intrinsic drug resistance. Molecular Cancer Therapeutics, 6(7), 2065–2072. 17620435 10.1158/1535-7163.MCT-06-0766

[jev212070-bib-0018] Barok, M. , Joensuu, H. , & Isola, J. (2014). Trastuzumab emtansine: Mechanisms of action and drug resistance. Breast Cancer Research, 16(2), 209. 24887180 10.1186/bcr3621 PMC4058749

[jev212070-bib-0019] Barok, M. , Le Joncour, V. , Martins, A. , Isola, J. , Salmikangas, M. , Laakkonen, P. , & Joensuu, H. (2020). ARX788, a novel anti‐HER2 antibody‐drug conjugate, shows anti‐tumor effects in preclinical models of trastuzumab emtansine‐resistant HER2‐positive breast cancer and gastric cancer. Cancer Letters, 473:156–163. 31904483 10.1016/j.canlet.2019.12.037

[jev212070-bib-0020] Barok, M. , Puhka, M. , Vereb, G. , Szollosi, J. , Isola, J. , & Joensuu, H. (2018). Cancer‐derived exosomes from HER2‐positive cancer cells carry trastuzumab‐emtansine into cancer cells leading to growth inhibition and caspase activation. Bmc Cancer [Electronic Resource], 18(1), 504. W. Robey, Ierano, Zhan, & E. Bates, 2011 10.1186/s12885-018-4418-2 PMC5930687 29720111

[jev212070-bib-0021] Barok, M. , Tanner, M. , Köninki, K. , & Isola, J. (2011). Trastuzumab‐DM1 causes tumour growth inhibition by mitotic catastrophe in trastuzumab‐resistant breast cancer cells in vivo. Breast Cancer Research, 13(2), R46. 21510863 10.1186/bcr2868 PMC3219209

[jev212070-bib-0022] Battke, C. , Ruiss, R. , Welsch, U. , Wimberger, P. , Lang, S. , Jochum, S. , & Zeidler, R. (2011). Tumour exosomes inhibit binding of tumour‐reactive antibodies to tumour cells and reduce ADCC. Cancer Immunology, Immunotherapy, 60(5), 639–648. 21293856 10.1007/s00262-011-0979-5 PMC11029199

[jev212070-bib-0023] Bebawy, M. , Combes, V. , Lee, E. , Jaiswal, R. , Gong, J. , Bonhoure, A. , & Grau, G. E. R. (2009). Membrane microparticles mediate transfer of P‐glycoprotein to drug sensitive cancer cells. Leukemia, 23(9), 1643–1649. 19369960 10.1038/leu.2009.76

[jev212070-bib-0024] Beck, A. , Goetsch, L. , Dumontet, C. , & Corvaïa, N. (2017). Strategies and challenges for the next generation of antibody‐drug conjugates. Nature Reviews Drug Discovery, 16(5), 315–337. 28303026 10.1038/nrd.2016.268

[jev212070-bib-0025] Becker, A. , Thakur, B. K. , Weiss, J. M. , Kim, H. S. , Peinado, H. , & Lyden, D. (2016). Extracellular Vesicles in Cancer: Cell‐to‐Cell Mediators of Metastasis. Cancer Cell, 30(6), 836–848. 27960084 10.1016/j.ccell.2016.10.009 PMC5157696

[jev212070-bib-0026] Bendell, J. , Moore, K. , Qin, A. , Johnson, D. , Schindler, J. , Papadopoulos, K. , & Tolcher, A. W. (2010). A phase I study of IMGN388, an antibody drug conjugate targeting av integrin, in patients with solid tumors. European Journal of Cancer Supplements, 8(7), 152.

[jev212070-bib-0027] Birrer, M. J. , Moore, K. N. , Betella, I. , & Bates, R. C. (2019). Antibody‐Drug Conjugate‐Based Therapeutics: State of the Science. Journal of the National Cancer Institute, 111(6), 538–549. 30859213 10.1093/jnci/djz035

[jev212070-bib-0028] Boukouris, S. , & Mathivanan, S. (2015). Exosomes in bodily fluids are a highly stable resource of disease biomarkers. Proteomics – Clinical Applications, 9(3‐4), 358–367. 25684126 10.1002/prca.201400114 PMC5502131

[jev212070-bib-0029] Buschow, S. I. , van Balkom, B. W. , Aalberts, M. , Heck, A. J. , Wauben, M. , & Stoorvogel, W. (2010). MHC class II‐associated proteins in B‐cell exosomes and potential functional implications for exosome biogenesis. Immunology and Cell Biology, 88(8), 851–856. 20458337 10.1038/icb.2010.64

[jev212070-bib-0030] Cardillo, T. M. , Govindan, S. V. , Sharkey, R. M. , Trisal, P. , & Goldenberg, D. M. (2011). Humanized anti‐Trop‐2 IgG‐SN‐38 conjugate for effective treatment of diverse epithelial cancers: Preclinical studies in human cancer xenograft models and monkeys. Clinical Cancer Research, 17(10), 3157–3169. 21372224 10.1158/1078-0432.CCR-10-2939 PMC10766325

[jev212070-bib-0031] Challita‐Eid, P. M. , Satpayev, D. , Yang, P. , An, Z. , Morrison, K. , Shostak, Y. , Raitano, A. , Nadell, R. , Liu, W. , Lortie, D. R. , Capo, L. , Verlinsky, A. , Leavitt, M. , Malik, F. , Aviña, H. , Guevara, C. I. , Dinh, N. , Karki, S. , Anand, B. S. , … Stover, D. R. (2016). Enfortumab vedotin antibody‐drug conjugate targeting nectin‐4 is a highly potent therapeutic agent in multiple preclinical cancer Models. Cancer Research, 76(10), 3003–3013. 27013195 10.1158/0008-5472.CAN-15-1313

[jev212070-bib-0032] Chang, C.‐H. , Wang, Y. , Zalath, M. , Liu, D. , Cardillo, T M. , & Goldenberg, D M. (2016). Combining ABCG2 inhibitors with IMMU‐132, an anti‐trop‐2 antibody conjugate of SN‐38, overcomes resistance to SN‐38 in breast and gastric cancers. Molecular Cancer Therapeutics, 15(8), 1910–1919. 27207776 10.1158/1535-7163.MCT-16-0219

[jev212070-bib-0033] Chau, C. H. , Steeg, P. S. , & Figg, W. D. (2019). Antibody‐drug conjugates for cancer. Lancet, 394(10200), 793–804. 31478503 10.1016/S0140-6736(19)31774-X

[jev212070-bib-0034] Chen, R. , Herrera, A. F. , Hou, J. , Chen, Lu , Wu, J. , Guo, Y. , Synold, T. W. , Ngo, V. N. , Puverel, S. , Mei, M. , Popplewell, L. , Yi, S. , Song, J. Y. , Tao, S. , Wu, X. , Chan, W. C. , Forman, S. J. , Kwak, L. W. , Rosen, S. T. , & Newman, E. M. (2020). Inhibition of MDR1 overcomes resistance to brentuximab vedotin in Hodgkin lymphoma. Clinical Cancer Research, 26(5), 1034–1044. 31811017 10.1158/1078-0432.CCR-19-1768 PMC7056527

[jev212070-bib-0035] Ciardiello, C. , Cavallini, L. , Spinelli, C. , Yang, J. , Reis‐Sobreiro, M. , de Candia, P. , Minciacchi, V. R. , & Di Vizio, D. (2016). Focus on Extracellular Vesicles: New Frontiers of Cell‐to‐Cell Communication in Cancer. International Journal of Molecular Sciences, 17(2), 175. 26861306 10.3390/ijms17020175 PMC4783909

[jev212070-bib-0036] Ciravolo, V. , Huber, V. , Ghedini, G C. , Venturelli, E. , Bianchi, F. , Campiglio, M. , Morelli, D. , Villa, A. , Mina, P. D. , Menard, S. , Filipazzi, P. , Rivoltini, L. , Tagliabue, E. , & Pupa, S M. et al. (2002). Gemtuzumab ozogamicin, a potent and selective anti‐CD33 antibody‐calicheamicin conjugate for treatment of acute myeloid leukemia. Bioconjugate Chemistry, 13(1), 658–667. 10.1021/bc010021y 11792178

[jev212070-bib-0037] Ciravolo, V. , Huber, V. , Ghedini, G. C. , Venturelli, E. , Bianchi, F. , Campiglio, M. , Morelli, D. , Villa, A. , Della Mina, P. , Menard, S. , Filipazzi, P. , Rivoltini, L. , Tagliabue, E. , & Pupa, S M. (2012). Potential role of HER2‐overexpressing exosomes in countering trastuzumab‐based therapy. Journal of Cellular Physiology, 227(2), 658–667. 21465472 10.1002/jcp.22773

[jev212070-bib-0038] Coats, S. , Williams, M. , Kebble, B. , Dixit, R. , Tseng, L. , Yao, N.‐S. , Tice, D. A. , & Soria, J.‐C. (2019). Antibody‐drug conjugates: future directions in clinical and translational strategies to improve the therapeutic index. Clinical Cancer Research, 25(18), 5441–5448. 30979742 10.1158/1078-0432.CCR-19-0272

[jev212070-bib-0039] Collins, D. , Bossenmaier, B. , Kollmorgen, G. , & Niederfellner, G. (2019). Acquired Resistance to Antibody‐Drug Conjugates. Cancers (Basel), 11(3), 394. 10.3390/cancers11030394 PMC6468698 30897808

[jev212070-bib-0040] Corcoran, C. , Rani, S. , O'brien, K. , O'neill, A. , Prencipe, M. , Sheikh, R. , Webb, G. , Mcdermott, R. , Watson, W. , Crown, J. , & O'driscoll, L. (2012). Docetaxel‐resistance in prostate cancer: Evaluating associated phenotypic changes and potential for resistance transfer via exosomes. PLoS One, 7(12), e50999. 23251413 10.1371/journal.pone.0050999 PMC3519481

[jev212070-bib-0041] Dan, N. , Setua, S. , Kashyap, V. K. , Khan, S. , Jaggi, M. , Yallapu, M. M. , & Chauhan, S. C. (2018). Antibody‐drug conjugates for cancer therapy: Chemistry to clinical implications. Pharmaceuticals (Basel), 11(2). 10.3390/ph11020032 PMC6027311 29642542

[jev212070-bib-0042] De Bono, J. S. , Concin, N. , Hong, D. S. , Thistlethwaite, F. C. , Machiels, J.‐P. , Arkenau, H.‐T. , Plummer, R. , Jones, R. H. , Nielsen, D. , Windfeld, K. , Ghatta, S. , Slomovitz, B. M. , Spicer, J. F. , Yachnin, J. , Ang, J. E. , Mau‐Sørensen, P. M. , Forster, M. D. , Collins, D. , Dean, E. , … Lassen, U. (2019). Tisotumab vedotin in patients with advanced or metastatic solid tumours (InnovaTV 201): A first‐in‐human, multicentre, phase 1–2 trial. The Lancet Oncology, 20(3), 383–393. 30745090 10.1016/S1470-2045(18)30859-3

[jev212070-bib-0043] Dijoseph, J F. , Armellino, D C. , Boghaert, E R. , Khandke, K. , Dougher, M M. , Sridharan, L. , Kunz, A. , Hamann, P R. , Gorovits, B. , Udata, C. , Moran, J K. , Popplewell, A G. , Stephens, S. , Frost, P. , & Damle, N K. (2004). Antibody‐targeted chemotherapy with CMC‐544: A CD22‐targeted immunoconjugate of calicheamicin for the treatment of B‐lymphoid malignancies. Blood, 103(5), 1807–1814. 14615373 10.1182/blood-2003-07-2466

[jev212070-bib-0044] Donaghy, H. (2016). Effects of antibody, drug and linker on the preclinical and clinical toxicities of antibody‐drug conjugates. MAbs, 8(4), 659–671. 27045800 10.1080/19420862.2016.1156829 PMC4966843

[jev212070-bib-0045] Dornan, D. , Bennett, F. , Chen, Y. , Dennis, M. , Eaton, D. , Elkins, K. , French, D. , Go, M. A. T. , Jack, A. , Junutula, J. R. , Koeppen, H. , Lau, J. , Mcbride, J. , Rawstron, A. , Shi, X. , Yu, N. , Yu, S.‐F. , Yue, P. , Zheng, B. , … Polson, A G. (2009). Therapeutic potential of an anti‐CD79b antibody‐drug conjugate, anti‐CD79b‐vc‐MMAE, for the treatment of non‐Hodgkin lymphoma. Blood, 114(13), 2721–2729. 19633198 10.1182/blood-2009-02-205500

[jev212070-bib-0046] European Medicines Agency , (2019). Committee for Medicinal Products for Human Use, Assessment report, Polivy. EMA/CHMP/690748/2019

[jev212070-bib-0047] Federici, C. , Petrucci, F. , Caimi, S. , Cesolini, A. , Logozzi, M. , Borghi, M. , D'ilio, S. , Lugini, L. , Violante, N. , Azzarito, T. , Majorani, C. , Brambilla, D. , & Fais, S. (2014). Exosome release and low pH belong to a framework of resistance of human melanoma cells to cisplatin. PLoS One, 9(2), e88193. 24516610 10.1371/journal.pone.0088193 PMC3916404

[jev212070-bib-0048] Gamis, A. S. , Alonzo, T. A. , Meshinchi, S. , Sung, L. , Gerbing, R. B. , Raimondi, S. C. , Hirsch, B. A. , Kahwash, S. B. , Heerema‐McKenney, A. , Winter, L. , Glick, K. , Davies, S. M. , Byron, P. , Smith, F. O. , & Aplenc, R. (2014). Gemtuzumab ozogamicin in children and adolescents with de novo acute myeloid leukemia improves event‐free survival by reducing relapse risk: Results from the randomized phase III Children's Oncology Group trial AAML0531. Journal of Clinical Oncology, 32(27), 3021–3032. 25092781 10.1200/JCO.2014.55.3628 PMC4162498

[jev212070-bib-0049] García‐Alonso, S. , Ocaña, A. , & Pandiella, A. (2018). Resistance to antibody‐drug conjugates. Cancer Research, 78(9), 2159–2165. 29653942 10.1158/0008-5472.CAN-17-3671

[jev212070-bib-0050] Garnier, D. , Magnus, N. , Lee, T. H. , Bentley, V. , Meehan, B. , Milsom, C. , Montermini, L. , Kislinger, T. , & Rak, J. (2012). Cancer cells induced to express mesenchymal phenotype release exosome‐like extracellular vesicles carrying tissue factor. Journal of Biological Chemistry, 287(52), 43565–43572. 10.1074/jbc.M112.401760 PMC3527943 23118232

[jev212070-bib-0051] Garrett, M. , Ruiz‐Garcia, A. , Parivar, K. , Hee, B. , & Boni, J. (2019). Population pharmacokinetics of inotuzumab ozogamicin in relapsed/refractory acute lymphoblastic leukemia and non‐Hodgkin lymphoma. Journal of Pharmacokinetics and Pharmacodynamics, 46(3), 211–222. 30859374 10.1007/s10928-018-9614-9 PMC6529376

[jev212070-bib-0052] Goss, G. D. , Vokes, E. E. , Gordon, M. S. , Gandhi, L. , Papadopoulos, K. P. , Rasco, D. W. , Fischer, J. S. , Chu, K. L. , Ames, W. W. , Mittapalli, R. K. , Lee, Ho‐J. , Zeng, J. , Roberts‐Rapp, L. A. , Loberg, L. I. , Ansell, P. J. , Reilly, E. B. , Ocampo, C. J. , Holen, K. D. , & Tolcher, A. W. (2018). Efficacy and safety results of depatuxizumab mafodotin (ABT‐414) in patients with advanced solid tumors likely to overexpress epidermal growth factor receptor. Cancer, 124(10), 2174–2183. 29533458 10.1002/cncr.31304 PMC5969257

[jev212070-bib-0053] Hamblett, K. J. , Kozlosky, C. J. , Siu, S. , Chang, W. S. , Liu, H. , Foltz, I. N. , Trueblood, E. S. , Meininger, D. , Arora, T. , Twomey, B. , Vonderfecht, S. L. , Chen, Q. , Hill, J. S. , & Fanslow, W. C. (2015). AMG 595, an Anti‐EGFRvIII antibody‐drug conjugate, induces potent antitumor activity against EGFRvIII‐expressing glioblastoma. Molecular Cancer Therapeutics, 14(7), 1614–1624. 25931519 10.1158/1535-7163.MCT-14-1078

[jev212070-bib-0054] Hansen, H. P. , Trad, A. , Dams, M. , Zigrino, P. , Moss, M. , Tator, M. , Schön, G. , Grenzi, P. C. , Bachurski, D. , Aquino, B. , Dürkop, H. , Reiners, K. S. , Von Bergwelt‐Baildon, M. , Hallek, M. , Grötzinger, J. , Engert, A. , Leme, A. F. P. , & Von Strandmann, E. P. (2016). CD30 on extracellular vesicles from malignant Hodgkin cells supports damaging of CD30 ligand‐expressing bystander cells with Brentuximab‐Vedotin, in vitro. Oncotarget, 7(21), 30523–30535. 27105521 10.18632/oncotarget.8864 PMC5058698

[jev212070-bib-0055] Hiddemann, W. , Kneba, M. , Dreyling, M. , Schmitz, N. , Lengfelder, E. , Schmits, R. , Reiser, M. , Metzner, B. , Harder, H. , Hegewisch‐Becker, S. , Fischer, T. , Kropff, M. , Reis, H.‐E. , Freund, M. , Wörmann, B. , Fuchs, R. , Planker, M. , Schimke, J. , Eimermacher, H. , … Unterhalt, M. (2005). Frontline therapy with rituximab added to the combination of cyclophosphamide, doxorubicin, vincristine, and prednisone (CHOP) significantly improves the outcome for patients with advanced‐stage follicular lymphoma compared with therapy with CHOP alone: Results of a prospective randomized study of the German Low‐Grade Lymphoma Study Group. Blood, 106(12), 3725–3732. 16123223 10.1182/blood-2005-01-0016

[jev212070-bib-0056] Horwitz, S. , O'connor, O. A. , Pro, B. , Illidge, T. , Fanale, M. , Advani, R. , Bartlett, N. L. , Christensen, J. H. , Morschhauser, F. , Domingo‐Domenech, E. , Rossi, G. , Kim, W. S. , Feldman, T. , Lennard, A. , Belada, D. , Illés, Á. , Tobinai, K. , Tsukasaki, K. , Yeh, S.‐P. , … Zinzani, P. L. (2019). Brentuximab vedotin with chemotherapy for CD30‐positive peripheral T‐cell lymphoma (ECHELON‐2): A global, double‐blind, randomised, phase 3 trial. Lancet, 393(10168), 229–240. 30522922 10.1016/S0140-6736(18)32984-2 PMC6436818

[jev212070-bib-0057] Hoshino, A. , Costa‐Silva, B. , Shen, T.‐L. , Rodrigues, G. , Hashimoto, A. , Tesic Mark, M. , Molina, H. , Kohsaka, S. , Di Giannatale, A. , Ceder, S. , Singh, S. , Williams, C. , Soplop, N. , Uryu, K. , Pharmer, L. , King, T. , Bojmar, L. , Davies, A E. , Ararso, Y. , … Lyden, D. (2015). Tumour exosome integrins determine organotropic metastasis. Nature, 527(7578), 329–335. 26524530 10.1038/nature15756 PMC4788391

[jev212070-bib-0058] Hunter, F. W. , Barker, H. R. , Lipert, B. , Rothe, F. , Gebhart, G. , Piccart‐Gebhart, M. J. , Sotiriou, C. , & Jamieson, S. M. F. (2020). Mechanisms of resistance to trastuzumab emtansine (T‐DM1) in HER2‐positive breast cancer. British Journal of Cancer, 122(5), 603–612. 31839676 10.1038/s41416-019-0635-y PMC7054312

[jev212070-bib-0059] Hurvitz, S A. , Dirix, L. , Kocsis, J. , Bianchi, G V. , Lu, J. , Vinholes, J. , Guardino, E. , Song, C. , Tong, B. , Ng, V. , Chu, Yu‐W. , & Perez, E A. (2013). Phase II randomized study of trastuzumab emtansine versus trastuzumab plus docetaxel in patients with human epidermal growth factor receptor 2‐positive metastatic breast cancer. Journal of Clinical Oncology, 31(9), 1157–1163. 23382472 10.1200/JCO.2012.44.9694

[jev212070-bib-0060] Jakhar, R. , & Crasta, K. (2019). Exosomes as emerging pro‐tumorigenic mediators of the senescence‐associated secretory phenotype. International Journal of Molecular Sciences, 20(10), 2547. 10.3390/ijms20102547 PMC6566274 31137607

[jev212070-bib-0061] Jen, E. Y. , Ko, C. W. , Lee, J. E. , Del Valle, P. L. , Aydanian, A. , Jewell, C. , Norsworthy, K. J. , Przepiorka, D. , Nie, L. , Liu, J. , Sheth, C. M. , Shapiro, M. , Farrell, A. T. , & Pazdur, R. (2018). FDA Approval: Gemtuzumab ozogamicin for the treatment of adults with newly diagnosed CD33‐positive acute myeloid leukemia. Clinical Cancer Research, 24(14), 3242–3246. 29476018 10.1158/1078-0432.CCR-17-3179

[jev212070-bib-0062] Junttila, T. T. , Li, G. , Parsons, K. , Phillips, G. L. , & Sliwkowski, M. X. (2011). Trastuzumab‐DM1 (T‐DM1) retains all the mechanisms of action of trastuzumab and efficiently inhibits growth of lapatinib insensitive breast cancer. Breast Cancer Research and Treatment, 128(2), 347–356. 20730488 10.1007/s10549-010-1090-x

[jev212070-bib-0063] Kalluri, R. (2016). The biology and function of exosomes in cancer. The Journal of Clinical Investigation, 126(4), 1208–1215. 27035812 10.1172/JCI81135 PMC4811149

[jev212070-bib-0064] Kantarjian, H. M. , Deangelo, D. J. , Stelljes, M. , Martinelli, G. , Liedtke, M. , Stock, W. , Gökbuget, N. , O'brien, S. , Wang, K. , Wang, T. , Paccagnella, M. L. , Sleight, B. , Vandendries, E. , & Advani, A. S. (2016). Inotuzumab Ozogamicin versus Standard Therapy for Acute Lymphoblastic Leukemia. New England Journal of Medicine, 375(8), 740–753. 10.1056/NEJMoa1509277 PMC5594743 27292104

[jev212070-bib-0065] Khongorzul, P. , Ling, C. J. , Khan, F. U. , Ihsan, A. U. , & Zhang, J. (2020). Antibody‐Drug Conjugates: A Comprehensive Review. Molecular Cancer Research, 18(1), 3–19. 31659006 10.1158/1541-7786.MCR-19-0582

[jev212070-bib-0066] King, G. T. , Eaton, K. D. , Beagle, B. R. , Zopf, C. J. , Wong, G. Y. , Krupka, H. I. , Hua, S. Y. , Messersmith, W. A. , & El‐Khoueiry, A. B. (2018). A phase 1, dose‐escalation study of PF‐06664178, an anti‐Trop‐2/Aur0101 antibody‐drug conjugate in patients with advanced or metastatic solid tumors. Investigational New Drugs, 36(5), 836–847. 29333575 10.1007/s10637-018-0560-6 PMC7519583

[jev212070-bib-0067] Koch, R. , Aung, T. , Vogel, D. , Chapuy, B. , Wenzel, D. , Becker, S. , Sinzig, U. , Venkataramani, V. , Von Mach, T. , Jacob, R. , Truemper, L. , & Wulf, G G. (2016). Nuclear Trapping through inhibition of exosomal export by indomethacin increases cytostatic efficacy of doxorubicin and pixantrone. Clinical Cancer Research, 22(2), 395–404. 26369630 10.1158/1078-0432.CCR-15-0577

[jev212070-bib-0068] Köninki, K. , Barok, M. , Tanner, M. , Staff, S. , Pitkänen, J. , Hemmilä, P. , Ilvesaro, J. , & Isola, J. (2010). Multiple molecular mechanisms underlying trastuzumab and lapatinib resistance in JIMT‐1 breast cancer cells. Cancer Letters, 294(2), 211–219.Wortzel, Dror, Kenific, & Lyden, 2019 20193978 10.1016/j.canlet.2010.02.002

[jev212070-bib-0069] Kosaka, N. , Yoshioka, Y. , Fujita, Yu , & Ochiya, T. (2016). Versatile roles of extracellular vesicles in cancer. The Journal of clinical investigation, 126(4), 1163–1172. 26974161 10.1172/JCI81130 PMC4811151

[jev212070-bib-0070] Le Joncour, V. , Martins, A. , Puhka, M. , Isola, J. , Salmikangas, M. , Laakkonen, P. , Joensuu, H. , & Barok, M. (2019). A novel anti‐HER2 antibody‐drug conjugate XMT‐1522 for HER2‐positive breast and gastric cancers resistant to trastuzumab emtansine. Molecular Cancer Therapeutics, 18(10), 1721–1730. 31292166 10.1158/1535-7163.MCT-19-0207

[jev212070-bib-0071] Lewis Phillips, G. D. , Li, G. , Dugger, D. L. , Crocker, L. M. , Parsons, K. L. , Mai, E. , Blättler, W. A. , Lambert, J. M. , Chari, R. V. J. , Lutz, R J. , Wong, W. L. T. , Jacobson, F. S. , Koeppen, H. , Schwall, R. H. , Kenkare‐Mitra, S. R. , Spencer, S. D. , & Sliwkowski, M. X. (2008). Targeting HER2‐positive breast cancer with trastuzumab‐DM1, an antibody‐cytotoxic drug conjugate. Cancer research, 68(22), 9280–9290. 19010901 10.1158/0008-5472.CAN-08-1776

[jev212070-bib-0072] Li, G. , Guo, J. , Shen, B.‐Q. , Yadav, D. B. , Sliwkowski, M. X. , Crocker, L M. , Lacap, J A. , & Phillips, G D. L. (2018). Mechanisms of acquired resistance to trastuzumab emtansine in breast cancer cells. Molecular Cancer Therapeutics, 17(7), 1441–1453. 29695635 10.1158/1535-7163.MCT-17-0296

[jev212070-bib-0073] Linenberger, M L. , Hong, T. , Flowers, D. , Sievers, E. L. , Gooley, T. A. , Bennett, J. M. , Berger, M. S. , Leopold, L. H. , Appelbaum, F. R. , & Bernstein, I. D. (2001). Multidrug‐resistance phenotype and clinical responses to gemtuzumab ozogamicin. Blood, 98(4), 988–994. 11493443 10.1182/blood.v98.4.988

[jev212070-bib-0074] Linenberger, M. L. (2005). CD33‐directed therapy with gemtuzumab ozogamicin in acute myeloid leukemia: Progress in understanding cytotoxicity and potential mechanisms of drug resistance. Leukemia, 19(2), 176–182. 15592433 10.1038/sj.leu.2403598

[jev212070-bib-0075] Lonial, S. , Lee, H. C. , Badros, A. , Trudel, S. , Nooka, A. K. , Chari, A. , Abdallah, A. O. , Callander, N. , Lendvai, N. , Sborov, D. , Suvannasankha, A. , Weisel, K. , Karlin, L. , Libby, E. , Arnulf, B. , Facon, T. , Hulin, C. , Kortüm, K. M. , Rodríguez‐Otero, P. , … Cohen, A. D. (2020). Belantamab mafodotin for relapsed or refractory multiple myeloma (DREAMM‐2): A two‐arm, randomised, open‐label, phase 2 study. The Lancet Oncology, 21(2), 207–221. 31859245 10.1016/S1470-2045(19)30788-0

[jev212070-bib-0076] Lu, H. , Bowler, N. , Harshyne, L. A. , Craig Hooper, D. , Krishn, S. R. , Kurtoglu, S. , Fedele, C. , Liu, Q. , Tang, H. Y. , & Kossenkov, A. V. , Kelly, W. K. , Wang, K. , Kean, R. B. , Weinreb, P. H. , Yu, L. , Dutta, A. , Fortina, P. , Ertel, A. , Stanczak, M. , … Languino, L. R. (2018). Exosomal alphavbeta6 integrin is required for monocyte M2 polarization in prostate cancer. Matrix Biology, 70, 20–35. 29530483 10.1016/j.matbio.2018.03.009 PMC6081240

[jev212070-bib-0077] Maacha, S. , Bhat, A A. , Jimenez, L. , Raza, A. , Haris, M. , Uddin, S. , & Grivel, J.‐C. (2019). Extracellular vesicles‐mediated intercellular communication: Roles in the tumor microenvironment and anti‐cancer drug resistance. Molecular Cancer [Electronic Resource], 18(1), 55. 10.1186/s12943-019-0965-7 PMC6441157 30925923

[jev212070-bib-0078] Melo, S. A. , Luecke, L. B. , Kahlert, C. , Fernandez, A. F. , Gammon, S. T. , Kaye, J. , LeBleu, V. S. , Mittendorf, E. A. , Weitz, J. , Rahbari, N. , Reissfelder, C. , Pilarsky, C. , Fraga, M. F. , Piwnica‐Worms, D. , & Kalluri, R. (2015). Glypican‐1 identifies cancer exosomes and detects early pancreatic cancer. Nature, 523(7559), 177–182. 26106858 10.1038/nature14581 PMC4825698

[jev212070-bib-0079] Modi, S. , Saura, C. , Yamashita, T. , Park, Y. H. , Kim, S.‐B. , Tamura, K. , Andre, F. , Iwata, H. , Ito, Y. , Tsurutani, J. , Sohn, J. , Denduluri, N. , Perrin, C. , Aogi, K. , Tokunaga, E. , Im, S.‐A. , Lee, K. S. , Hurvitz, S A. , Cortes, J. , … Krop, I. (2020). Trastuzumab deruxtecan in previously treated her2‐positive breast cancer. New England Journal of Medicine, 382(7), 610–621. 10.1056/NEJMoa1914510 PMC7458671 31825192

[jev212070-bib-0080] Moquist, P. N. , Bovee, T. D. , Waight, A. B. , Mitchell, J. A. , Miyamoto, J. B. , Mason, M. L. , Emmerton, K. K. , Stevens, N. , Balasubramanian, C. , Simmons, J. K. , Lyon, R. P. , Senter, P. D. , & Doronina, S. O. (2020). Novel auristatins with high bystander and cytotoxic activities in drug‐efflux positive tumor models. Molecular Cancer Therapeutics molcanther.0618.2020, Dec 7;molcanther.0618. 10.1158/1535-7163.MCT-20-0618 33288628

[jev212070-bib-0081] Mulcahy, L. A. , Pink, R. C. , & Carter, D. R. (2014). Routes and mechanisms of extracellular vesicle uptake. Journal of Extracell Vesicles, *4*, 3. 10.3402/jev.v3.24641 PMC4122821 25143819

[jev212070-bib-0082] Nabhan, C. , & Rosen, S T. (2014). Chronic lymphocytic leukemia: A clinical review. JAMA, 312(21), 2265–2276. 25461996 10.1001/jama.2014.14553

[jev212070-bib-0083] Namee, N Mc , & O'driscoll, L. (2018). Extracellular vesicles and anti‐cancer drug resistance. Biochim Biophys Acta Rev Cancer, 1870(2), 123–136. 30003999 10.1016/j.bbcan.2018.07.003

[jev212070-bib-0084] Norsworthy, K. J. , Ko, C. W. , Lee, J. E. , Liu, J. , John, C. S. , Przepiorka, D. , Farrell, A. T. , & Pazdur, R. (2018). FDA Approval Summary: Mylotarg for treatment of patients with relapsed or refractory CD33‐positive acute myeloid leukemia. The Oncologist, 23(9), 1103–1108. 29650683 10.1634/theoncologist.2017-0604 PMC6192608

[jev212070-bib-0085] O'Connor‐McCourt, M. , Koropatnick, J. , Maleki, S. , Figueredo, R. , Tikhomirov, I. , & Jaramillo, M. (2016). Development of AVID100, a novel antibody–drug conjugate for the treatment of EGFR expressing solid tumors. European Journal of Cancer, Volume 69, Supplement 1, S147.

[jev212070-bib-0086] Ogitani, Y. , Aida, T. , Hagihara, K. , Yamaguchi, J. , Ishii, C. , Harada, N. , Soma, M. , Okamoto, H. , Oitate, M. , Arakawa, S. , Hirai, T. , Atsumi, R. , Nakada, T. , Hayakawa, I. , Abe, Y. , & Agatsuma, T. (2016). DS‐8201a, A novel HER2‐targeting ADC with a novel DNA topoisomerase I inhibitor, demonstrates a promising antitumor efficacy with differentiation from T‐DM1. Clinical Cancer Research, 22(20), 5097–5108. 27026201 10.1158/1078-0432.CCR-15-2822

[jev212070-bib-0087] Oksvold, M. P. , Kullmann, A. , Forfang, L. , Kierulf, B. , Li, Mu , Brech, A. , Vlassov, A. V. , Smeland, E. B. , Neurauter, A. , & Pedersen, K. W. (2014). Expression of B‐cell surface antigens in subpopulations of exosomes released from B‐cell lymphoma cells. Clinical Therapeutics, 36(6), 847–862.e1 e841. 24952935 10.1016/j.clinthera.2014.05.010

[jev212070-bib-0088] Parslow, A. , Parakh, S. , Lee, F.‐T. , Gan, H. , & Scott, A. (2016). Antibody‐drug conjugates for cancer therapy. Biomedicines, 4(3), 14. 10.3390/biomedicines4030014 PMC5344263 28536381

[jev212070-bib-0089] Paul, M. R. , Wong, V. , Aristizabal, P. , & Kuo, D. J. (2019). Treatment of recurrent refractory pediatric pre‐B Acute lymphoblastic leukemia using inotuzumab ozogamicin monotherapy resulting in CD22 antigen expression loss as a mechanism of therapy resistance. Journal of Pediatric Hematology/Oncology, 41(8), e546–e549. 30807395 10.1097/MPH.0000000000001440 PMC7216755

[jev212070-bib-0090] Peinado, H. , Zhang, H. , Matei, I R. , Costa‐Silva, B. , Hoshino, A. , Rodrigues, G. , Psaila, B. , Kaplan, R N. , Bromberg, J F. , Kang, Y. , Bissell, M J. , Cox, T R. , Giaccia, A J. , Erler, J T. , Hiratsuka, S. , Ghajar, C M. , & Lyden, D. (2017). Pre‐metastatic niches: Organ‐specific homes for metastases. Nature Reviews Cancer, 17(5), 302–317. 28303905 10.1038/nrc.2017.6

[jev212070-bib-0091] Perez‐Amill, L. , Suñe, G. , Antoñana‐Vildosola, A. , Castella, M. , Najjar, A. , Bonet, J. , Fernández‐Fuentes, N. , Inogés, S. , López, A. , Bueno, C. , Juan, M. , Urbano‐Ispizua, Á. , & Martín‐Antonio, B. (2020). Preclinical development of a humanized chimeric antigen receptor against B cell maturation antigen for multiple myeloma. Haematologica 173–184, 10.3324/haematol.2019.228577 PMC7776337 31919085

[jev212070-bib-0092] Pitt, J M. , Kroemer, G. , & Zitvogel, L. (2016). Extracellular vesicles: Masters of intercellular communication and potential clinical interventions. The Journal of clinical investigation, 126(4), 1139–1143. 27035805 10.1172/JCI87316 PMC4811136

[jev212070-bib-0093] Prince, H. M. , Kim, Y. H. , Horwitz, S. M. , Dummer, R. , Scarisbrick, J. , Quaglino, P. , Zinzani, P. L. , Wolter, P. , Sanches, J. A. , Ortiz‐Romero, P. L. , Akilov, O. E. , Geskin, L. , Trotman, J. , Taylor, K. , Dalle, S. , Weichenthal, M. , Walewski, J. , Fisher, D. , Dréno, B. , … Duvic, M. (2017). Brentuximab vedotin or physician's choice in CD30‐positive cutaneous T‐cell lymphoma (ALCANZA): An international, open‐label, randomised, phase 3, multicentre trial. Lancet, 390(10094), 555–566. 28600132 10.1016/S0140-6736(17)31266-7

[jev212070-bib-0094] Puhka, M. , Takatalo, M. , Nordberg, M.‐E. , Valkonen, S. , Nandania, J. , Aatonen, M. , Yliperttula, M. , Laitinen, S. , Velagapudi, V. , Mirtti, T. , Kallioniemi, O. , Rannikko, A. , Siljander, P. R.‐.M. , & Af Hällström, T. M. (2017). Metabolomic profiling of extracellular vesicles and alternative normalization methods reveal enriched metabolites and strategies to study prostate cancer‐related changes. Theranostics, 7(16), 3824–3841. 29109780 10.7150/thno.19890 PMC5667407

[jev212070-bib-0095] Raab‐Westphal, S. , Marshall, J. F. , & Goodman, S. L. (2017). Integrins as therapeutic targets: Successes and cancers. Cancers (Basel), 9(9). 10.3390/cancers9090110 PMC5615325 28832494

[jev212070-bib-0096] Ratajczak, J. , Wysoczynski, M. , Hayek, F. , Janowska‐Wieczorek, A. , & Ratajczak, M. Z. (2006). Membrane‐derived microvesicles: Important and underappreciated mediators of cell‐to‐cell communication. Leukemia, 20(9), 1487–1495. 16791265 10.1038/sj.leu.2404296

[jev212070-bib-0097] Ricart, A. D. (2011). Antibody‐drug conjugates of calicheamicin derivative: Gemtuzumab ozogamicin and inotuzumab ozogamicin. Clinical Cancer Research, 17(20), 6417–6427. 22003069 10.1158/1078-0432.CCR-11-0486

[jev212070-bib-0098] Rosenberg, J. E. , O'donnell, P. H. , Balar, A. V. , Mcgregor, B. A. , Heath, E. I. , Yu, E. Y. , Galsky, M. D. , Hahn, N. M. , Gartner, E. M. , Pinelli, J. M. , Liang, S.‐Y. , Melhem‐Bertrandt, A. , & Petrylak, D. P. (2019). Pivotal trial of enfortumab vedotin in urothelial carcinoma after platinum and anti‐programmed death 1/programmed death ligand 1 therapy. Journal of Clinical Oncology, 37(29), 2592–2600. 31356140 10.1200/JCO.19.01140 PMC6784850

[jev212070-bib-0099] Rosenberg, J. , Sridhar, S. S. , Zhang, J. , Smith, D. , Ruether, D. , Flaig, T. W. , Baranda, J. , Lang, J. , Plimack, E. R. , Sangha, R. , Heath, E. I. , Merchan, J. , Quinn, D. I. , Srinivas, S. , Milowsky, M. , Wu, C. , Gartner, E. M. , Zuo, P. , Melhem‐Bertrandt, A. , & Petrylak, D. P. (2020). EV‐101: A phase I study of single‐agent enfortumab vedotin in patients with nectin‐4‐positive solid tumors, including metastatic urothelial carcinoma. Journal of Clinical Oncology, 38(10), 1041–1049. 32031899 10.1200/JCO.19.02044 PMC7106979

[jev212070-bib-0100] Safaei, R. , Larson, B. J. , Cheng, T. C. , Gibson, M. A. , Otani, S. , Naerdemann, W. , & Howell, S. B. (2005). Abnormal lysosomal trafficking and enhanced exosomal export of cisplatin in drug‐resistant human ovarian carcinoma cells. Molecular Cancer Therapeutics, 4(10), 1595–1604. 16227410 10.1158/1535-7163.MCT-05-0102

[jev212070-bib-0101] Sehn, L. H. , Herrera, A. F. , Flowers, C. R. , Kamdar, M. K. , McMillan, A. , Hertzberg, M. , Assouline, S. , Kim, T. M. , Kim, W. S. , Ozcan, M. , Hirata, J. , Penuel, E. , Paulson, J. N. , Cheng, J. , Ku, G. , & Matasar, M. J. (2020). Polatuzumab Vedotin in Relapsed or Refractory Diffuse Large B‐Cell Lymphoma. Journal of Clinical Oncology, 38(2), 155–165. 31693429 10.1200/JCO.19.00172 PMC7032881

[jev212070-bib-0102] Setiady, Y. Y. , Dong, L. , Skaletskaya, A. , Pinkas, J. , Lutz, R. J. , Lambert, J. M. , & Chittenden, T. (2014). IMGN289, an EGFR‐targeting antibody‐drug conjugate, is effective against tumor cells that are resistant to EGFR tyrosine kinase inhibitors. In: Proceedings of the 105th Annual Meeting of the American Association for Cancer Research; 2014 Apr 5–9; San Diego, CA Philadelphia (PA): AACR; Cancer Research;74(19 Suppl), ABstract nr 4513 2014.

[jev212070-bib-0103] Sharkey, R. M. , McBride, W. J. , Cardillo, T. M. , Govindan, S. V. , Wang, Y. , Rossi, E. A. , Chang, C. H. , & Goldenberg, D. M. (2015). Enhanced Delivery of SN‐38 to human tumor xenografts with an anti‐trop‐2‐SN‐38 antibody conjugate (sacituzumab govitecan). Clinical Cancer Research, 21(22), 5131–5138. 26106073 10.1158/1078-0432.CCR-15-0670

[jev212070-bib-0104] Shedden, K. , Xie, X. T. , Chandaroy, P. , Chang, Y. T. , & Rosania, G. R. (2003). Expulsion of small molecules in vesicles shed by cancer cells: Association with gene expression and chemosensitivity profiles. Cancer Research, 63(15), 4331–4337. 12907600

[jev212070-bib-0105] Slamon, D. J. , Leyland‐Jones, B. , Shak, S. , Fuchs, H. , Paton, V. , Bajamonde, A. , Fleming, T. , Eiermann, W. , Wolter, J. , Pegram, M. , Baselga, J. , & Norton, L. (2001). Use of chemotherapy plus a monoclonal antibody against HER2 for metastatic breast cancer that overexpresses HER2. New England Journal of Medicine, 344:783–792. 10.1056/NEJM200103153441101 11248153

[jev212070-bib-0106] Smith, M. R. (2003). Rituximab (monoclonal anti‐CD20 antibody): Mechanisms of action and resistance. Oncogene, 22(47), 7359–7368. 14576843 10.1038/sj.onc.1206939

[jev212070-bib-0107] Strebhardt, K. , & Ullrich, A. (2008). Paul Ehrlich's magic bullet concept: 100 years of progress. Nature Reviews Cancer, 8(6), 473–480. 18469827 10.1038/nrc2394

[jev212070-bib-0108] Szczepanski, M. J. , Szajnik, M. , Welsh, A. , Whiteside, T. L. , & Boyiadzis, M. (2011). Blast‐derived microvesicles in sera from patients with acute myeloid leukemia suppress natural killer cell function via membrane‐associated transforming growth factor‐beta1. Haematologica, 96(9), 1302–1309. 21606166 10.3324/haematol.2010.039743 PMC3166100

[jev212070-bib-0109] Tai, Y.‐T. , Mayes, P. A. , Acharya, C. , Zhong, M. Y. , Cea, M. , Cagnetta, A. , Craigen, J. , Yates, J. , Gliddon, L. , Fieles, W. , Hoang, B. , Tunstead, J. , Christie, A. L. , Kung, A. L. , Richardson, P. , Munshi, N. C. , & Anderson, K. C. (2014). Novel anti‐B‐cell maturation antigen antibody‐drug conjugate (GSK2857916) selectively induces killing of multiple myeloma. Blood, 123(20), 3128–3138. 24569262 10.1182/blood-2013-10-535088 PMC4023420

[jev212070-bib-0110] Takegawa, N. , Nonagase, Y. , Yonesaka, K. , Sakai, K. , Maenishi, O. , Ogitani, Y. , Tamura, T. , Nishio, K. , Nakagawa, K. , & Tsurutani, J. (2017). DS‐8201a, a new HER2‐targeting antibody‐drug conjugate incorporating a novel DNA topoisomerase I inhibitor, overcomes HER2‐positive gastric cancer T‐DM1 resistance. International Journal of Cancer, 141(8), 1682–1689. 28677116 10.1002/ijc.30870

[jev212070-bib-0111] Takeshita, A. (2013). Efficacy and resistance of gemtuzumab ozogamicin for acute myeloid leukemia. International Journal of Hematology, 97(6), 703–716. 23709007 10.1007/s12185-013-1365-1

[jev212070-bib-0112] Takeshita, A. , Shinjo, K. , Yamakage, N. , Ono, T. , Hirano, I. , Matsui, H. , Shigeno, K. , Nakamura, S. , Tobita, T. , Maekawa, M. , Ohnishi, K. , Sugimoto, Y. , Kiyoi, H. , Naoe, T. , & Ohno, R. (2009). CMC‐544 (inotuzumab ozogamicin) shows less effect on multidrug resistant cells: Analyses in cell lines and cells from patients with B‐cell chronic lymphocytic leukaemia and lymphoma. British Journal of Haematology, 146(1), 34–43. 19388933 10.1111/j.1365-2141.2009.07701.x

[jev212070-bib-0113] Tian, T. , Zhu, Y.‐L. , Hu, F‐Hu , Wang, Y.‐Y. , Huang, N.‐P. , & Xiao, Z.‐D. (2013). Dynamics of exosome internalization and trafficking. Journal of Cellular Physiology, 228(7), 1487–1495. 23254476 10.1002/jcp.24304

[jev212070-bib-0114] Torreggiani, E. , Roncuzzi, L. , Perut, F. , Zini, N. , & Baldini, N. (2016). Multimodal transfer of MDR by exosomes in human osteosarcoma. International Journal of Oncology, 49(1), 189–196. 27176642 10.3892/ijo.2016.3509

[jev212070-bib-0115] Trerotola, M. , Ganguly, K. K. , Fazli, L. , Fedele, C. , Lu, H. , Dutta, A. , Liu, Q. , De Angelis, T. , Riddell, L. W. , Riobo, N. A. , Gleave, M. E., Zoubeidi, A., Pestell, R. G., Altieri, D. C., & Languino, L. R. (2015). Trop‐2 is up‐regulated in invasive prostate cancer and displaces FAK from focal contacts. Oncotarget, 6(16), 14318–14328. 26015409 10.18632/oncotarget.3960 PMC4546469

[jev212070-bib-0116] Valadi, H. , Ekström, K. , Bossios, A. , Sjöstrand, M. , Lee, J. J. , & Lötvall, J. O. (2007). Exosome‐mediated transfer of mRNAs and microRNAs is a novel mechanism of genetic exchange between cells. Nature Cell Biology, 9(6), 654–659. 17486113 10.1038/ncb1596

[jev212070-bib-0117] Van Niel, G. , D'angelo, G. , & Raposo, G. (2018). Shedding light on the cell biology of extracellular vesicles. Nature Reviews Molecular Cell Biology, 19(4), 213–228. 29339798 10.1038/nrm.2017.125

[jev212070-bib-0118] Varughese, J. , Cocco, E. , Bellone, S. , Bellone, M. , Todeschini, P. , Carrara, L. , Schwartz, P. E. , Rutherford, T. J. , Pecorelli, S. , & Santin, A. D. (2011). High‐grade, chemotherapy‐resistant primary ovarian carcinoma cell lines overexpress human trophoblast cell‐surface marker (Trop‐2) and are highly sensitive to immunotherapy with hRS7, a humanized monoclonal anti‐Trop‐2 antibody. Gynecologic Oncology, 122(1), 171–177. 21453957 10.1016/j.ygyno.2011.03.002 PMC3104081

[jev212070-bib-0119] Verma, S. , Miles, D. , Gianni, L. , Krop, I. E. , Welslau, M. , Baselga, J. , Pegram, M. , Oh, Do‐Y. , Diéras, V. , Guardino, E. , Fang, L. , Lu, M. W. , Olsen, S. , & Blackwell, K. (2012). Trastuzumab emtansine for HER2‐positive advanced breast cancer. New England Journal of Medicine, 367, 1783–1791. 10.1056/NEJMoa1209124 PMC5125250 23020162

[jev212070-bib-0120] Von Minckwitz, G. , Huang, C.‐S. , Mano, M. S. , Loibl, S. , Mamounas, E. P. , Untch, M. , Wolmark, N. , Rastogi, P. , Schneeweiss, A. , Redondo, A. , Fischer, H. H. , Jacot, W. , Conlin, A. K. , Arce‐Salinas, C. , Wapnir, I. L. , Jackisch, C. , Digiovanna, M. P. , Fasching, P. A. , Crown, J. P. , … Geyer, C. E. (2019). Trastuzumab emtansine for residual invasive HER2‐positive breast cancer. New England Journal of Medicine, 380(7), 617–628. 10.1056/NEJMoa1814017 30516102

[jev212070-bib-0121] W. Robey, R. , Ierano, C. , Zhan, Z. , & E. Bates, S. (2011). The challenge of exploiting ABCG2 in the clinic. Current Pharmaceutical Biotechnology, 12(4), 595–608. 21118093 10.2174/138920111795163913 PMC3091815

[jev212070-bib-0122] Wolska‐Washer, A. , & Robak, T. (2019). Safety and tolerability of antibody‐drug conjugates in cancer. Drug Safety, 42(2), 295–314. 30649747 10.1007/s40264-018-0775-7 PMC6399172

[jev212070-bib-0123] Wortzel, I. , Dror, S. , Kenific, C. M. , & Lyden, D. (2019). Exosome‐mediated metastasis: communication from a distance. Developmental Cell, 49(3), 347–360. 31063754 10.1016/j.devcel.2019.04.011

[jev212070-bib-0124] Xu, R.‐h. , Qiu M.‐Z. , Zhang, Y. , Wei, X.‐L. , & Hu, C. (2020). First‐in‐human dose‐escalation study of anti‐EGFR ADC MRG003 in patients with relapsed/refractory solid tumors. Journal of Clinical Oncology, v38 n15_suppl (20200520): 3550 ASCO.

[jev212070-bib-0125] Xu, Y. , & Villalona‐Calero, M. A. (2002). Irinotecan: Mechanisms of tumor resistance and novel strategies for modulating its activity. Annals of Oncology, 13(12), 1841–1851. 12453851 10.1093/annonc/mdf337

[jev212070-bib-0126] Younes, A. , Bartlett, N. L. , Leonard, J. P. , Kennedy, D. A. , Lynch, C. M. , Sievers, E. L. , & Forero‐Torres, A. (2010). Brentuximab vedotin (SGN‐35) for relapsed CD30‐positive lymphomas. New England Journal of Medicine, 363(19), 1812–1821. 10.1056/NEJMoa1002965 21047225

[jev212070-bib-0127] Yu, M. , Ocana, A. , & Tannock, I F. (2013). Reversal of ATP‐binding cassette drug transporter activity to modulate chemoresistance: Why has it failed to provide clinical benefit? Cancer and Metastasis Reviews, 32(1‐2), 211–227. 23093326 10.1007/s10555-012-9402-8

[jev212070-bib-0128] Yu, S.‐F. , Zheng, B. , Go, M. , Lau, J. , Spencer, S. , Raab, H. , Soriano, R. , Jhunjhunwala, S. , Cohen, R. , Caruso, M. , Polakis, P. , Flygare, J. , & Polson, A G. (2015). A Novel Anti‐CD22 Anthracycline‐Based Antibody‐Drug Conjugate (ADC) that overcomes resistance to auristatin‐based ADCs. Clinical Cancer Research, 21(14), 3298–3306. 25840969 10.1158/1078-0432.CCR-14-2035

[jev212070-bib-0129] Zhang, F.‐F. , Zhu, Yi‐F. , Zhao, Q.‐N. , Yang, D.‐T. , Dong, Ye‐P. , Jiang, Li , Xing, W.‐X. , Li, X.‐Y. , Xing, H. , Shi, M. , Chen, Y. , Bruce, I C. , Jin, J. , & Ma, X. (2014). Microvesicles mediate transfer of P‐glycoprotein to paclitaxel‐sensitive A2780 human ovarian cancer cells, conferring paclitaxel‐resistance. European Journal of Pharmacology, 738, 83–90. 24877693 10.1016/j.ejphar.2014.05.026

[jev212070-bib-0130] Zhang, H. , Deng, T. , Liu, R. , Bai, M. , Zhou, L. , Wang, X. , Li, S. , Wang, X. , Yang, H. , Li, J. , Ning, T., Huang, D., Li, H., Zhang, L., Ying, G., & Ba, Y. (2017). Exosome‐delivered EGFR regulates liver microenvironment to promote gastric cancer liver metastasis. Nature communications, 8, 15016. 10.1038/ncomms15016 PMC5394240 28393839

[jev212070-bib-0131] Zhao, H. , Yang, L. , Baddour, J. , Achreja, A. , Bernard, V. , Moss, T. , Marini, J. C. , Tudawe, T. , Seviour, E. G. , San Lucas, F. A. , Alvarez, H. , Gupta, S. , Maiti, S. N. , Cooper, L. , Peehl, D. , Ram, P. T. , Maitra, A. , & Nagrath, D. (2016). Tumor microenvironment derived exosomes pleiotropically modulate cancer cell metabolism. Elife, 5, e10250. 26920219 10.7554/eLife.10250 PMC4841778

