# Supplementary material for: Extracellular vesicles as modifiers of antibody‐drug conjugate efficacy
Source: J Extracell Vesicles. 2021 Feb 13;10(4):e12070. doi: 10.1002/jev2.12070 (PMC7881363; doi:10.1002/jev2.12070)
Supplement: Supplementary file 1 — Supplementary Table 1: Antibody‐drug conjugates (ADC) and their targets expressed on extracellular vesicles (EVs) [file JEV2-10-e12070-s001.docx]

| **ADC target detected on EVs** | **ADC / Developer** | **Linker-payload** | **Mechanism of action of the payload** | **Indication (stage)** | **Clinical trial** |  | **ADC target detected on EVs** | **Source of EVs** | **Method for target detection on EVs** | **Target localization on EVs** |
| --- | --- | --- | --- | --- | --- | --- | --- | --- | --- | --- |
| **AXL** | CAB-AXL-ADC (BA3011) / BioAtla [1] | Undisclosed linker-MMAE | Microtubule inhibitor | Multiple solid tumors (phase I-II) | NCT03425279 |  | **AXL** | Human melanoma and BC cells, serum of metastatic melanoma patients | nPLEX, Wb [2] | Surface |
|  | HuMax-Axl-ADC / Genmab [3] | vc-MMAE | Microtubule inhibitor | Multiple solid tumors (phase I-II) | NCT02988817 |  |  | Human cerebrospinal fluid | Wb [4] | N.D. |
| **BCMA** | AMG 224 / Amgen [5] | Undisclosed linker-DM1 | Microtubule inhibitor | MM (phase I) | NCT02561962 |  | **BCMA** | Human MM cells | IF [6] | Surface |
|  | Belantamab mafodotin (GSK2857916) / GlaxoSmithKline [7] | mc-MMAF | Microtubule inhibitor | Approved for MM | NCT04246047 |  |  |  |  |  |
|  | CC-99712 / Celgene | Undisclosed | Undisclosed | MM (phase I) | NCT04036461 |  |  | Human MM cells | Proteomics [8] | N.D. |
| **CD19** | Coltuximab ravtansine (SAR3419) / ImmunoGen [9] | spdb-DM4 | Microtubule inhibitor | ALL, DLBCL, NHL (phase II, discontinued) | NCT01472887 |  | **CD19** | Human B-cell lymphoma cells | FC, Wb [10] | Surface |
|  | Denintuzumab mafodotin (SGNCD19A) / Seattle Genetics [11] | mc-MMAF | Microtubule inhibitor | ALL, DLBCL, NHL (phase I-II, discontinued) | NCT02855359 |  |  | Plasma of CLL patients | DotScan [12] | N.D. |
|  | huB4-DGN462 / ImmunoGen [13] | sulfo-spdb-DGN462 | DNA alkylator | B-cell malignancies (preclinical) | – |  |  | Human CLL cells | FC [14] | Surface |
|  |  |  |  |  |  |  |  | Human RBCUs | FC [15] | Surface |
|  | Loncastuximab tesirine (ADCT-402) / ADC Therapeutics [16] | PEG8-va-SG3199 (PBD) | DNA alkylator | DLBCL (phase III) | NCT04384484 |  |  | Human B-cell lymphoma cells, serum of CC and GC patients | FC [17] | Surface |
|  | SGN-CD19B / Seattle Genetics [18] | mc-va-PBD | DNA alkylator | DLBCL, NHL (phase 1) | NCT02702141 |  |  | Serum of patients with B cell malignancies | FC [19] | Surface |
| **CD22 (Siglec-2)** | Inotuzumab ozogamicin (Besponsa, CMC-544) / Wyeth/Pfizer [20, 21] | AcBut-CalichDMH (derivative of calicheamicin) | DNA double strand breaker | Approved for  ALL | NCT01564784 |  | **CD22 (Siglec-2)** | **Mouse** B-cell lymphoma cells | FC [22] | Surface |
|  | Pinatuzumab vedotin (DCDT2980S) / Genentech/Roche [23] | mc-vc-PABC-MMAE | Microtubule inhibitor | DLBCL, NHL (phase II, discontinued) | NCT01691898 |  |  |  |  |  |
| **CD30** | Brentuximab vedotin (Adcetris, SGN-35) / Seattle Genetics [24, 25] | vc-MMAE | Microtubule inhibitor | Approved for  HL, SALCL, PCALCL, MF, PTCL | NCT01712490 |  | **CD30** | Human HL cells | FC, Wb [26] | Surface |
|  | F0002-ADC (anti-CD30-MCC-DM1) / Shanghai Fudan-Zhangjiang Bio-Pharmaceutical [27] | SMCC-DM1 | Microtubule inhibitor | CD30+ hematologic malignancies (phase I) | NCT03894150 |  |  | Human HL cells | FC [28] | Surface |
|  |  |  |  |  |  |  |  | Serum of HL patients | FC [19] | Surface |
| **CD33** | AVE9633 (huMy9-6-DM4) /  ImmunoGen/Sanofi [29] | SPDB-DM4 | Microtubule inhibitor | AML (phase I, discontinued) | NCT00543972 |  | **CD33** | Serum of AML patients | FC [30] | Surface |
|  | Gemtuzumab ozogamicin (Mylotarg, CMA-676) / Wyeth/Pfizer [31-33] | AcBut-calicheamicin | DNA double strand breaker | Approved for AML | NCT00927498 |  |  |  |  |  |
|  | IMGN779 (ImmunoGen) [34] | sulfo-SPDB-DGN462 | DNA-alkylator | AML (phase I, discontinued) | NCT02674763 |  |  |  |  |  |
|  | Vadastuximab talirine (SGN-CD33A) / Seattle Genetics [35] | mc-va-SGD1882 (PBD) | DNA alkylator | AML (phase III, discontinued) | NCT02785900 |  |  |  |  |  |
| **CD37** | AGS67E / Agensys/Astellas [36] | mc-vc-pabc-MMAE | Microtubule inhibitor | Lymphoid malignancies (phase I) | NCT02175433 |  | **CD37** | Human B-cell lymphoma cells | FC, Wb [10] | Surface |
|  |  |  |  |  |  |  |  | Plasma of CLL patients | DotScan [12] | N.D. |
|  | Naratuximab emtansine (IMGN529) / ImmunoGen [37] | SMCC-DM1 | Microtubule inhibitor | CLL, NHL (phase I) | NCT01534715 |  |  | Human CLL cells | FC [14] | Surface |
| **CD56** | Lorvotuzumab mertansine (IMGN901, huN901-SPP-DM1, BB-10901) / ImmunoGen [38] | SPP-DM1 | Microtubule inhibitor | Multiple solid tumors, MM, (phase II, discontinued) | NCT02452554 |  | **CD56** | Human NK cells, plasma of healthy donors | FC, immuno-EM [39] | Surface |
| **CD66e (CEACAM5** | Labetuzumab govitecan (IMMU-130) / Immunomedics [40] | CL2A-SN38 (active metabolite of irinotecan) | Topoisomerase I inhibitor | MCC (phase I-II) | NCT01270698 |  | **CD66e (CEACAM5)** | Human colorectal cancer cells | DotScan [12] | N.D. |
|  | SAR408701 / Sanofi [41, 42] | SPDB-DM4 | Microtubule inhibitor | Multiple solid tumors  (phase III) | NCT04154956 |  |  | Human colon cancer cells | Wb [43] | N.D. |
| **CD70 (CD27L, TNFSF7)** | AMG 172 / Amgen [44] | SMCC-DM1 | Microtubule inhibitor | RCC (phase I, discontinued) | NCT01497821 |  | **CD70 (CD27L, TNFSF7)** | Human malignant mesothelioma cells | Proteomics, Wb [45] | N.D. |
|  | BMS-936561 (MDX-1203, αCD70_MED‐A) / Bristol-Myers Squibb [46] | vc-MED-A (carbamate prodrug of duocarmycin derivative) | DNA alkylator | RCC, NHL (phase I, discontinued) | NCT00944905 |  |  |  |  |  |
|  | SGN-CD70A / Seattle Genetics [47] | va-SGD1882 (PBD) | DNA alkylator | RCC (phase I, discontinued) | NCT02216890 |  |  | Plasma of HNC patients | Wb [48] | N.D. |
|  | Vorsetuzumab mafodotin (SGN-75) / Seattle Genetics [49] | mc-MMAF | Microtubule inhibitor | NHL, RCC (phase I, discontinued) | NCT01015911 |  |  |  |  |  |
| **CD79b** | Polatuzumab vedotin (Polivy, DCDS4501A) / Genentech/ Roche [50, 51] | vc–MMAE | Microtubule inhibitor | Approved for DLBCL | NCT02257567 |  | **CD79b** | EB virus-transformed human B-cells | Proteomics [52] | N.D. |
| **CD117 (KIT)** | LOP628 / Novartis [53] | SMCC-DM1 | Microtubule inhibitor | AML, solid tumors (phase I, discontinued) | NCT02221505 |  | **CD117 (KIT)** | Human GIST cells | FC, Wb [54] | Surface |
|  |  |  |  |  |  |  |  | Serum of AML patients | FC [30] | Surface |
| **CD123** | IMGN632 / ImmunoGen [55] | Undisclosed peptide linker-MIP | DNA alkylator | ALL, AML, BPDCN (Phase I-II) | NCT03386513 |  | **CD123** | Plasma of AML patients | Wb [56] | N.D. |
|  | SGN-CD123A / Seattle Genetics [57] | mc-va-SGD1882 (PBD) | DNA alkylator | AML (phase I, discontinued) | NCT02848248 |  |  |  |  |  |
| **CD142 (Tissue factor)** | Tisotumab vedotin (HuMax-TF-ADC) / Genmab [58] | vc-MMAE | Microtubule inhibitor | Multiple solid tumors (phase II) | NCT03485209 |  | **CD142 (Tissue factor)** | Human RBCUs | FC [15] | Surface |
|  |  |  |  |  |  |  |  | Human BC and PC cells | Wb [59] | N.D. |
|  |  |  |  |  |  |  |  | Human epidermoid carcinoma cells | Wb [60] | N.D. |
| **CD138 (Syndecan-1)** | Indatuximab ravtansine (BT-062) / Biotest [61] | SPDB-DM4 | Microtubule inhibitor | MM (phase I- II) | NCT01638936 |  | **CD138 (Syndecan-1)** | Human bladder cancer cells | Proteomics [62] | N.D. |
|  |  |  |  |  |  |  |  | Human myeloma cells, serum of MM patients | FC, Wb [63] | Surface |
| **EGFR** | AVID-100 / Forbius [64] | SMCC-DM1 | Microtubule inhibitor | TNBC, HNSCC, NSCLC (phase I-II) | NCT03094169 |  | **EGFR** | Human GC cells, serum of GC patients | Wb [65] | N.D. |
|  | Depatuxizumab mafodotin* (ABT-414) / AbbVie [66] | mc-MMAF | Microtubule inhibitor | GM and solid tumors (phase III) | NCT03419403 |  |  | Human colorectal cancer cells | Wb [67] | N.D. |
|  |  |  |  |  |  |  |  | Human bladder cancer cells | Proteomics [62] | N.D. |
|  | Laprituximab emtansine (IMGN289) / ImmunoGen [68] | SMCC-DM1 | Microtubule inhibitor | Solid tumors (phase I, discontinued) | NCT01963715 |  |  | Human prostate cancer cells, serum/plasma of PC patients | ELISA, Wb [69] | N.D. |
|  | MRG003 / Shanghai Miracogen [70] | Undisclosed linker-MMAE | Microtubule inhibitor | Solid tumors (phase I) | CTR20180310 |  |  | Human epidermoid carcinoma cells | Wb [60] | N.D. |
|  | Serclutamab talirine (ABBV-321) / AbbVie [71] | mc-va-PBD | DNA alkylator | Solid tumors (phase I) | NCT03234712 |  |  | Human epidermoid cancer cells | Wb [72] | N.D. |
|  |  |  |  |  |  |  |  | Human BC cells | FC [73] | Surface |
| **EGFRvIII** | AMG 595 / Amgen [74] | SMCC-DM1 | Microtubule inhibitor | Recurrent GM (phase I, discontinued) | NCT01475006 |  | **EGFRvIII** | Human glioma cells | Wb [75] | N.D. |
|  | Depatuxizumab mafodotin* (ABT-414) / AbbVie [66] | mc-MMAF | Microtubule inhibitor | GM and solid tumors (phase III) | NCT03419403 |  |  |  |  |  |
| **Ephrin-A4** | PF-06647263 / Pfizer [76] | hydrazone-calicheamicin | DNA double strand breaker | TNBC, OC (phase I, discontinued) | NCT02078752 |  | **Ephrin-A4** | Human glioblastoma cells, **mouse** cortical neurons | Proteomics, Wb [77] | N.D. |
| **FGFR3** | LY3076226 / Eli Lilly [78] | sulfo-SPDB-DM4 | Microtubule inhibitor | Advanced metastatic cancers (phase I) | NCT02529553 |  | **FGFR3** | Human urine | Proteomics [79] | N.D. |
|  |  |  |  |  |  |  |  |  |  |  |
| **Glypican-1** | GPC1-ADC / Kochi University, Japan) [80, 81] | mc-vc-PABC-MMAF | Microtubule inhibitor | PC, UCC (preclinical) | – |  | **Glypican-1** | Human malignant mesothelioma cells | Proteomics, Wb [45] | N.D. |
|  |  |  |  |  |  |  |  | Serum of PC patients | FC, immuno-EM [82] | Surface |
| **HER2** | ADCT-502 / ADC Therapeutics [83] | Undisclosed linker-PBD | DNA alkylator | Solid tumors (phase I) discontinued | NCT03125200 |  | **HER2** | Human BC cells, serum of BC patients | FC, immuno-EM, Wb [73] | Surface |
|  | ALT-P7 (HM2-MMAE) / Alteogen [84] | Undisclosed linker-MMAE | Microtubule inhibitor | BC (phase I) | NCT03281824 |  |  |  |  |  |
|  | ARX-788 / Zhejiang Medicine/Ambrx [85] | Undisclosed linker- Amberstatin  269 (a proprietary version of MMAF) | Microtubule inhibitor | BC, BTC, GC (phase I) | NCT03255070 |  |  | Ascites of OC and BC patients | Wb [86] | N.D. |
|  | A166 / Klus Pharma [87] | vc-duostatin-5 (an MMAF derivative) | Microtubule inhibitor | Solid tumors (phase I) | NCT03602079 |  |  |  |  |  |
|  | BAT8001 / Bio-Thera Solutions) [88] | Undisclosed linker- maytansine derivative | Microtubule inhibitor | BC (phase III) | NCT04185649 |  |  |  |  |  |
|  | MEDI4276 / MedImmune [89, 90] | mc-AZ13599185 (tubulysin) | Microtubule inhibitor | BC, GC (phase I) discontinued | NCT02576548 |  |  |  |  |  |
|  | MRG002 / Shanghai Miracogen [91] | Undisclosed linker-MMAE | Microtubule inhibitor | Solid tumors (phase I) | CTR20181778 |  |  |  |  |  |
|  | PF‑06804103 (NG-HER2 ADC) / Pfizer [92] | vc-Aur0101 (auristatin) | Microtubule inhibitor | BC, GC (phase I) | [NCT03284723](http://clinicaltrials.gov/show/NCT03284723) |  |  | Human GC cells | FC, immuno-EM, [93] | Surface |
|  | RC48-ADC (hertuzumab-vc-MMAE) / RemeGen [94, 95] | vc-MMAE | Microtubule inhibitor | BC, GC, UC (phase II) | NCT03500380 |  |  | Human BC cells, ascites of OC patients | FC, Wb [96] | Surface |
|  | Trastuzumab emtansine (Kadcyla, T-DM1) / Genentech/Roche [97, 98] | SMCC-DM1 | Microtubule inhibitor | Approved for MBC, EBC | NCT01772472 |  |  | Human BC cells, ascites of OC patients, serum of BC patients | FC, immuno-EM, Wb [99] | Surface |
|  | Trastuzumab deruxtecan (Enhertu, DS-8201a) / Daiichi Sankyo [100] | mggpg-DX-8951 (exatecan derivative) | Topoisomerase I inhibitor | Approved for MBC | NCT03248492 |  |  | Human mesothelioma cells | Wb [101] | N.D. |
|  | Trastuzumab duocarmazine (SYD985, trastuzumab vc-seco-DUBA) / Synthon [102] | vc-seco-DUBA (duocarmycine) | DNA alkylator | MBC, MEC (phase III) | NCT03262935 |  |  | Human BC cells | FC, immuno-EM [103] | Surface |
|  | XMT-1522 / Mersana [93] | Fleximer polymer linker-AF-HPA | Microtubule inhibitor | BC, GC, NSCLC (phase I, discontinued) | NCT02952729 |  |  |  |  |  |
|  | ZW49 / Zymeworks [83] | Undisclosed linker-*N*-acyl sulfonamide auristatin | Microtubule inhibitor | Solid tumors (phase I) | NCT03821233 |  |  |  |  |  |
| **ITGαV** | IMGN388 / ImmunoGen [104, 105] | SPDB-DM4 | Microtubule inhibitor | Solid tumors, (phase I, discontinued) | NCT00721669 |  | **ITGαV** | Human PC, BC and melanoma cells, plasma of BC and PC patients with liver metastasis | ELISA, Proteomics [106] | N.D. |
|  |  |  |  |  |  |  |  | Human bladder cancer cells | Proteomics [62] | N.D. |
|  |  |  |  |  |  |  |  | Human colorectal cancer cells | Wb [67] | N.D. |
| **LAMP1** | SAR428926 / Sanofi [107] | SPDB-DM4 | Microtubule inhibitor | Solid tumors, TNBC (phase I) | NCT02575781 |  | **LAMP1** | Human mesothelioma cells | Wb [101] | N.D. |
|  |  |  |  |  |  |  |  | Human colorectal cancer cells | Wb [67] | N.D. |
| **Mesothelin** | Anetumab ravtansine (BAY 94-9343) / Bayer [108, 109] | SPDB-DM4 | Microtubule inhibitor | Solid tumors (phase II) | NCT03926143 |  | **Mesothelin** | Human mesothelioma cells | Proteomics, Wb [45] | N.D. |
|  | BMS-986148 / Bristol-Myers Squibb [110] | Undisclosed linker-tubulysin | Microtubule inhibitor | Solid tumors (phase I-II) | NCT02341625 |  |  | Human mesothelioma cells | Wb [101] | N.D. |
|  | RG7600 (DMOT4039A) / Genentech/Roche [111] | vc-MMAE | Microtubule inhibitor | OC, PC (phase I, discontinued) | NCT01469793 |  |  |  |  |  |
| **PSMA** | MEDI3726 (ADCT-401) / MedImmune/ADC Therapeutics [112] | PEG8-va-SG3199 (PBD) | DNA alkylator | PrC (phase I, discontinued) | NCT02991911 |  | **PSMA** | Human prostate cancer cells, plasma of PrC patients | EVs were isolated by magnetic beads conjugated with anti-PSMA antibody, Wb [113] | Surface |
|  | MLN2704 / Millenium/Takeda [114] | SPP-DM1 | Microtubule inhibitor | PrC (phase I, discontinued) | NCT00070837 |  |  |  |  |  |
|  | PSMA ADC / Progenics [115] | vc-MMAE | Microtubule inhibitor | GM, PrC (phase II) | NCT01695044 |  |  | Human PrC cells | Wb [116] | N.D. |
| **Trop-2** | BAT8003 / Bio-Thera Solutions) [117] | Undisclosed linker- maytansine derivative | Microtubule inhibitor | Solid tumors (phase I) | NCT03884517 |  | **Trop-2** | Human PrC cells | Wb [118] | N.D. |
|  | DS-1062a / Daiichi Sankyo [119] | Undisclosed linker-DXd (exatecan derivative) | Topoisomerase I inhibitor | NSCLC (phase I) | [NCT03401385](http://clinicaltrials.gov/show/NCT03401385) |  |  |  |  |  |
|  | PF-06664178 (RN927C) / Pfizer [120] | vc-PF-06380101(Aur0101, an (auristatin-based payload) | Microtubule inhibitor | Solid tumors (phase I, discontinued) | NCT02122146 |  |  |  |  |  |
|  | Sacituzumab govitecan (Trodelvy, IMMU-132) / Immunomedics [121, 122] | CL2A-SN38 (active metabolite of irinotecan) | Topoisomerase I inhibitor | Approved for MTNBC | NCT01631552 |  |  |  |  |  |
|  | SKB264 / KLUS Pharm [123] | Undisclosed linker- belotecan-derivative | Topoisomerase I inhibitor | Solid tumors (phase II) | NCT04152499 |  |  |  |  |  |

**Supplementary Table 1. Antibody-drug conjugates (ADC) and their targets expressed on extracellular vesicles (EVs)**

* Depatuxizumab mafodotin binds to a unique conformation of human EGFR that is exposed due to EGFR overexpression or a mutant form of EGFR, EGFRvIII [66].

**Abbreviations for Supplementary Table 1**

AcBut: 4-(4′-acetylphenoxy) butanoic acid

AF-HPA: Auristatin F-hydroxypropylamide

ALL: Acute lymphocytic leukemia

AML: Acute myeloid leukemia

BC: Breast cancer

BCMA: B-cell maturation antigen

BPDCN: Blastic plasmacytoid dendritic cell neoplasm

BTC: Biliary tract cancer

CalichDMH: N-acetyl-γ-calicheamicin dimethyl hydrazide

CC: Colon cancer

CEACAM5: Carcinoembryonic antigen-related cell adhesion molecule 5

CL2A: A proprietary, cleavable PEG8- and triazole-containing p-aminobenzyloxycarbonyl-peptide-maleimidocaproyl linker [124]

CLL: Chronic lymphocytic leukemia

DGN462: An indolinobenzodiazeprine pseudodimer [34]

DLBCL: Diffuse large B-cell lymphoma

DM1: Derivative of maytansine 1

DM4: Thiol-containing maytansinoid derivative

DUBA: Duocarmycin hydroxybenzamide azaindole

EB: Epstein-Barr virus

EBC: Early breast cancer

EGFR: Epidermal growth factor receptor

EGFRvIII: Epidermal growth factor receptor variant III

ELISA: Enzyme-linked immunosorbent assay

EM: Electron microscope

EV: Extracellular vesicle

FC: Flow cytometry

FGFR3: Fibroblast growth factor receptor-3

GC: Gastric cancer

GIST: Gastrointestinal stromal tumor

GM: Glioblastoma multiforme

HCC: Hepatocellular carcinoma

HER2: Human epidermal growth factor receptor-2

HL: Hodgkin lymphoma

HNC: Head and neck cancer

HNSCC: Head and neck squamous cell carcinoma

IF: Immunofluorescence

ITG: Integrin

MBC: Metastatic breast cancer

mc: Maleimidocaproyl

MCC: Metastatic colorectal cancer

mc-va: Maleimidocaproyl-valine-alanine

mc-vc-pabc: Maleimidocaproyl-valine-citrulline-p-aminobenzoyloxycarbonyl

mggpg: Maleimide glycine-glicyne-phenylalanine-glycine

MEC: Metastatic endometrial carcinoma

MF: Mycosis fungoides

MIP: Monoimine indolinobenzodiazepine pseudodimer

MM: Multiple myeloma

MMAE: Monomethyl auristatin E

MMAF: Monomethyl auristatin F

MPNST: Malignant peripheral nerve sheath tumor

MS: Mass spectrometry

MTNBC: Metastatic triple-negative breast cancer

NB: Neuroblastoma

N.D.: No data

NHL: Non-Hodgkin lymphoma

NSCLC: Non small cell lung cancer

nPLEX: Nano-plasmonic exosome assay

OC: Ovarian cancer

PABC: P-aminobenzyloxycarbonyl

PBD: Pyrrolobenzodiazepine

PC: Pancreatic cancer

PCALCL: Primary cutaneous anaplastic large cell lymphoma

PEG8: Polyethylene glycol 8

PPB: Pleuropulmonary blastoma

PrC: Prostate cancer

PSMA: Prostate-specific membrane antigen

PTCL: Peripheral T-cell lymphomas

RBCU: Red blood cell unit

RCC: Renal cell carcinoma

RMS: Rhabdomyosarcoma

SALCL: Systemic anaplastic large cell lymphoma

smcc: N-succinimidyl-4-(N-maleimidomethyl) cyclohexane-1-carboxylate

SN38: 7-ethyl-10-hydroxycamptothecin, an active metabolite of irinotecan

SPDB: *N*-succinimidyl-4-(2-pyridyldithio)butyrate

SPP: *N*-succinimidyl-3-(2-pyridyldithio)propionate

SS: Synovial sarcoma

TNBC: Triple negative breast cancer

Trop-2: Trophoblast cell-surface antigen 2

UC: Urothelial cancer

UCC: Uterine cervical cancer

va: Valine-alanine

vc: Valine-citrulline

Wb: Western blot

WT: Wilms tumor

**References**

1. Ahnert JR, Taylor MH, O'Reilly EM, Zhang J, Doebele RC, Ben Y, Sharp LL, Boyle WJ, Chang C, Frey G et al: A phase 1/2 dose-escalation and expansion study of a conditionally active anti-AXL humanized monoclonal antibody (BA3011) in patients with advanced solid tumors. Journal of Clinical Oncology 2018, 36.

2. Miller MA, Oudin MJ, Sullivan RJ, Wang SJ, Meyer AS, Im H, Frederick DT, Tadros J, Griffith LG, Lee H et al: Reduced Proteolytic Shedding of Receptor Tyrosine Kinases Is a Post-Translational Mechanism of Kinase Inhibitor Resistance. Cancer Discov 2016, 6(4):382-399.

3. Breij ECW, Verploegen S, Lingnau A, van den Brink EN, Janmaat M, Houtkamp M, Bleeker W, Satijn D, Parren P: Preclinical efficacy studies using HuMax-Axl-ADC, a novel antibody-drug conjugate targeting Axl-expressing solid cancers. Journal of Clinical Oncology 33, no 15_suppl (May 20, 2015) 3066-3066 2015.

4. Coulter ME, Dorobantu CM, Lodewijk GA, Delalande F, Cianferani S, Ganesh VS, Smith RS, Lim ET, Xu CS, Pang S et al: The ESCRT-III Protein CHMP1A Mediates Secretion of Sonic Hedgehog on a Distinctive Subtype of Extracellular Vesicles. Cell Rep 2018, 24(4):973-986 e978.

5. Lee HC, Raje NS, Landgren O, Upreti VV, Wang J, Avilion AA, Hu X, Rasmussen E, Ngarmchamnanrith G, Fujii H et al: Phase 1 study of the anti-BCMA antibody-drug conjugate AMG 224 in patients with relapsed/refractory multiple myeloma. Leukemia 2020.

6. Perez-Amill L, Sune G, Antonana-Vildosola A, Castella M, Najjar A, Bonet J, Fernandez-Fuentes N, Inoges S, Lopez A, Bueno C et al: Preclinical development of a humanized chimeric antigen receptor against B cell maturation antigen for multiple myeloma. Haematologica 2020.

7. Lonial S, Lee HC, Badros A, Trudel S, Nooka AK, Chari A, Abdallah AO, Callander N, Lendvai N, Sborov D et al: Belantamab mafodotin for relapsed or refractory multiple myeloma (DREAMM-2): a two-arm, randomised, open-label, phase 2 study. Lancet Oncol 2020, 21(2):207-221.

8. Hoelzinger DB, Quinton SJ, Walters DK, Tschumper RC, Jelinek DF: Proteomic and Biological Analysis of Myeloma Cell Derived Extracellular Vesicles. Blood (2018) 132 (Supplement 1): 5605.

9. Blanc V, Bousseau A, Caron A, Carrez C, Lutz RJ, Lambert JM: SAR3419: an anti-CD19-Maytansinoid Immunoconjugate for the treatment of B-cell malignancies. Clin Cancer Res 2011, 17(20):6448-6458.

10. Oksvold MP, Kullmann A, Forfang L, Kierulf B, Li M, Brech A, Vlassov AV, Smeland EB, Neurauter A, Pedersen KW: Expression of B-cell surface antigens in subpopulations of exosomes released from B-cell lymphoma cells. Clin Ther 2014, 36(6):847-862 e841.

11. Law C, Sutherland M, Miyamoto J, Hayes D, Duniho S, Boursalian T, Stone I, Jonas M, Smith L, Benjamin D: Preclinical characterization of an auristatin-based anti-CD19 drug conjugate, SGN-19A. In: Proceedings of the 102nd Annual Meeting of the American Association for Cancer Research; 2011 Apr 2-6; Orlando, FL Philadelphia (PA): AACR; Cancer Res 2011;71(8 Suppl):Abstract nr 625 2011.

12. Belov L, Matic KJ, Hallal S, Best OG, Mulligan SP, Christopherson RI: Extensive surface protein profiles of extracellular vesicles from cancer cells may provide diagnostic signatures from blood samples. J Extracell Vesicles 2016, 5:25355.

13. Hicks SW, Tarantelli C, Wilhem A, Gaudio E, Li M, Arribas AJ, Spriano F, Bordone R, Cascione L, Lai KC et al: The novel CD19-targeting antibody-drug conjugate huB4-DGN462 shows improved anti-tumor activity compared to SAR3419 in CD19-positive lymphoma and leukemia models. Haematologica 2019, 104(8):1633-1639.

14. Farahani M, Rubbi C, Liu L, Slupsky JR, Kalakonda N: CLL Exosomes Modulate the Transcriptome and Behaviour of Recipient Stromal Cells and Are Selectively Enriched in miR-202-3p. PLoS One 2015, 10(10):e0141429.

15. Danesh A, Inglis HC, Jackman RP, Wu S, Deng X, Muench MO, Heitman JW, Norris PJ: Exosomes from red blood cell units bind to monocytes and induce proinflammatory cytokines, boosting T-cell responses in vitro. Blood 2014, 123(5):687-696.

16. Zammarchi F, Corbett S, Adams L, Tyrer PC, Kiakos K, Janghra N, Marafioti T, Britten CE, Havenith CEG, Chivers S et al: ADCT-402, a PBD dimer-containing antibody drug conjugate targeting CD19-expressing malignancies. Blood 2018, 131(10):1094-1105.

17. Zhang F, Li R, Yang Y, Shi C, Shen Y, Lu C, Chen Y, Zhou W, Lin A, Yu L et al: Specific Decrease in B-Cell-Derived Extracellular Vesicles Enhances Post-Chemotherapeutic CD8(+) T Cell Responses. Immunity 2019, 50(3):738-750 e737.

18. Ryan MC, Palanca-Wessels MC, Schimpf B, Gordon KA, Kostner H, Meyer B, Yu C, Van Epps HA, Benjamin D: Therapeutic potential of SGN-CD19B, a PBD-based anti-CD19 drug conjugate, for treatment of B-cell malignancies. Blood 2017, 130(18):2018-2026.

19. Caivano A, Laurenzana I, De Luca L, La Rocca F, Simeon V, Trino S, D'Auria F, Traficante A, Maietti M, Izzo T et al: High serum levels of extracellular vesicles expressing malignancy-related markers are released in patients with various types of hematological neoplastic disorders. Tumour Biol 2015, 36(12):9739-9752.

20. Kantarjian HM, DeAngelo DJ, Stelljes M, Martinelli G, Liedtke M, Stock W, Gokbuget N, O'Brien S, Wang K, Wang T et al: Inotuzumab Ozogamicin versus Standard Therapy for Acute Lymphoblastic Leukemia. N Engl J Med 2016, 375(8):740-753.

21. DiJoseph JF, Armellino DC, Boghaert ER, Khandke K, Dougher MM, Sridharan L, Kunz A, Hamann PR, Gorovits B, Udata C et al: Antibody-targeted chemotherapy with CMC-544: a CD22-targeted immunoconjugate of calicheamicin for the treatment of B-lymphoid malignancies. Blood 2004, 103(5):1807-1814.

22. Ayre DC, Chute IC, Joy AP, Barnett DA, Hogan AM, Grull MP, Pena-Castillo L, Lang AS, Lewis SM, Christian SL: CD24 induces changes to the surface receptors of B cell microvesicles with variable effects on their RNA and protein cargo. Sci Rep 2017, 7(1):8642.

23. Li D, Poon KA, Yu SF, Dere R, Go M, Lau J, Zheng B, Elkins K, Danilenko D, Kozak KR et al: DCDT2980S, an anti-CD22-monomethyl auristatin E antibody-drug conjugate, is a potential treatment for non-Hodgkin lymphoma. Mol Cancer Ther 2013, 12(7):1255-1265.

24. Ansell SM: Brentuximab vedotin. Blood 2014, 124(22):3197-3200.

25. Horwitz S, O'Connor OA, Pro B, Illidge T, Fanale M, Advani R, Bartlett NL, Christensen JH, Morschhauser F, Domingo-Domenech E et al: Brentuximab vedotin with chemotherapy for CD30-positive peripheral T-cell lymphoma (ECHELON-2): a global, double-blind, randomised, phase 3 trial. Lancet 2019, 393(10168):229-240.

26. Hansen HP, Trad A, Dams M, Zigrino P, Moss M, Tator M, Schon G, Grenzi PC, Bachurski D, Aquino B et al: CD30 on extracellular vesicles from malignant Hodgkin cells supports damaging of CD30 ligand-expressing bystander cells with Brentuximab-Vedotin, in vitro. Oncotarget 2016, 7(21):30523-30535.

27. Shen Y, Yang T, Cao X, Zhang Y, Zhao L, Li H, Zhao T, Xu J, Zhang H, Guo Q et al: Conjugation of DM1 to anti-CD30 antibody has potential antitumor activity in CD30-positive hematological malignancies with lower systemic toxicity. MAbs 2019, 11(6):1149-1161.

28. Tosetti F, Vene R, Camodeca C, Nuti E, Rossello A, D'Arrigo C, Galante D, Ferrari N, Poggi A, Zocchi MR: Specific ADAM10 inhibitors localize in exosome-like vesicles released by Hodgkin lymphoma and stromal cells and prevent sheddase activity carried to bystander cells. Oncoimmunology 2018, 7(5):e1421889.

29. Lapusan S, Vidriales MB, Thomas X, de Botton S, Vekhoff A, Tang R, Dumontet C, Morariu-Zamfir R, Lambert JM, Ozoux ML et al: Phase I studies of AVE9633, an anti-CD33 antibody-maytansinoid conjugate, in adult patients with relapsed/refractory acute myeloid leukemia. Invest New Drugs 2012, 30(3):1121-1131.

30. Szczepanski MJ, Szajnik M, Welsh A, Whiteside TL, Boyiadzis M: Blast-derived microvesicles in sera from patients with acute myeloid leukemia suppress natural killer cell function via membrane-associated transforming growth factor-beta1. Haematologica 2011, 96(9):1302-1309.

31. Norsworthy KJ, Ko CW, Lee JE, Liu J, John CS, Przepiorka D, Farrell AT, Pazdur R: FDA Approval Summary: Mylotarg for Treatment of Patients with Relapsed or Refractory CD33-Positive Acute Myeloid Leukemia. Oncologist 2018, 23(9):1103-1108.

32. Hamann PR, Hinman LM, Hollander I, Beyer CF, Lindh D, Holcomb R, Hallett W, Tsou HR, Upeslacis J, Shochat D et al: Gemtuzumab ozogamicin, a potent and selective anti-CD33 antibody-calicheamicin conjugate for treatment of acute myeloid leukemia. Bioconjug Chem 2002, 13(1):47-58.

33. Ricart AD: Antibody-drug conjugates of calicheamicin derivative: gemtuzumab ozogamicin and inotuzumab ozogamicin. Clin Cancer Res 2011, 17(20):6417-6427.

34. Kovtun Y, Noordhuis P, Whiteman KR, Watkins K, Jones GE, Harvey L, Lai KC, Portwood S, Adams S, Sloss CM et al: IMGN779, a Novel CD33-Targeting Antibody-Drug Conjugate with DNA-Alkylating Activity, Exhibits Potent Antitumor Activity in Models of AML. Mol Cancer Ther 2018, 17(6):1271-1279.

35. Kung Sutherland MS, Walter RB, Jeffrey SC, Burke PJ, Yu C, Kostner H, Stone I, Ryan MC, Sussman D, Lyon RP et al: SGN-CD33A: a novel CD33-targeting antibody-drug conjugate using a pyrrolobenzodiazepine dimer is active in models of drug-resistant AML. Blood 2013, 122(8):1455-1463.

36. Pereira DS, Guevara CI, Jin L, Mbong N, Verlinsky A, Hsu SJ, Avina H, Karki S, Abad JD, Yang P et al: AGS67E, an Anti-CD37 Monomethyl Auristatin E Antibody-Drug Conjugate as a Potential Therapeutic for B/T-Cell Malignancies and AML: A New Role for CD37 in AML. Mol Cancer Ther 2015, 14(7):1650-1660.

37. Deckert J, Park PU, Chicklas S, Yi Y, Li M, Lai KC, Mayo MF, Carrigan CN, Erickson HK, Pinkas J et al: A novel anti-CD37 antibody-drug conjugate with multiple anti-tumor mechanisms for the treatment of B-cell malignancies. Blood 2013, 122(20):3500-3510.

38. Whiteman KR, Johnson HA, Mayo MF, Audette CA, Carrigan CN, LaBelle A, Zukerberg L, Lambert JM, Lutz RJ: Lorvotuzumab mertansine, a CD56-targeting antibody-drug conjugate with potent antitumor activity against small cell lung cancer in human xenograft models. MAbs 2014, 6(2):556-566.

39. Lugini L, Cecchetti S, Huber V, Luciani F, Macchia G, Spadaro F, Paris L, Abalsamo L, Colone M, Molinari A et al: Immune surveillance properties of human NK cell-derived exosomes. J Immunol 2012, 189(6):2833-2842.

40. Sharkey RM, Govindan SV, Cardillo TM, Donnell J, Xia J, Rossi EA, Chang CH, Goldenberg DM: Selective and Concentrated Accretion of SN-38 with a CEACAM5-Targeting Antibody-Drug Conjugate (ADC), Labetuzumab Govitecan (IMMU-130). Mol Cancer Ther 2018, 17(1):196-203.

41. Gazzah A, Stjepanovic N, Ryu MH, Tabernero J, Soria JC, Bedard P, Kang YK, Bahleda R, Guillemin-Paveau H, Henry C et al: First-in-human phase I trial of the anti-CEACAM5 antibody–drug conjugate SAR408701 in patients with advanced solid tumors (NCT02187848). European Journal of Cancer, Volume 69, Supplement 1, S14-S15, December 01, 2016 2016.

42. Decary S, Berne PF, Nicolazzi C, Lefebvre AM, Dabdoubi T, Cameron B, Rival P, Devaud C, Prades C, Bouchard H et al: Preclinical activity of SAR408701, a novel anti-CEACAM5-maytansinoid antibody-drug conjugate for the treatment of CEACAM5-positive epithelial tumors. Clin Cancer Res 2020.

43. Muturi HT, Dreesen JD, Nilewski E, Jastrow H, Giebel B, Ergun S, Singer BB: Tumor and endothelial cell-derived microvesicles carry distinct CEACAMs and influence T-cell behavior. PLoS One 2013, 8(9):e74654.

44. Massard C, Soria JC, Krauss J, Gordon M, Lockhart AC, Rasmussen E, Upreti VV, Patel S, Ngarmchamnanrith G, Henary H: First-in-human study to assess safety, tolerability, pharmacokinetics, and pharmacodynamics of the anti-CD27L antibody-drug conjugate AMG 172 in patients with relapsed/refractory renal cell carcinoma. Cancer Chemother Pharmacol 2019, 83(6):1057-1063.

45. Greening DW, Ji H, Chen M, Robinson BW, Dick IM, Creaney J, Simpson RJ: Secreted primary human malignant mesothelioma exosome signature reflects oncogenic cargo. Sci Rep 2016, 6:32643.

46. Wang H, Rangan VS, Sung MC, Passmore D, Kempe T, Wang X, Thevanayagam L, Pan C, Rao C, Srinivasan M et al: Pharmacokinetic characterization of BMS-936561, an anti-CD70 antibody-drug conjugate, in preclinical animal species and prediction of its pharmacokinetics in humans. Biopharm Drug Dispos 2016, 37(2):93-106.

47. Pal SK, Forero-Torres A, Thompson JA, Morris JC, Chhabra S, Hoimes CJ, Vogelzang NJ, Boyd T, Bergerot PG, Adashek JJ et al: A phase 1 trial of SGN-CD70A in patients with CD70-positive, metastatic renal cell carcinoma. Cancer 2019, 125(7):1124-1132.

48. Ludwig S, Floros T, Theodoraki MN, Hong CS, Jackson EK, Lang S, Whiteside TL: Suppression of Lymphocyte Functions by Plasma Exosomes Correlates with Disease Activity in Patients with Head and Neck Cancer. Clin Cancer Res 2017, 23(16):4843-4854.

49. Tannir NM, Forero-Torres A, Ramchandren R, Pal SK, Ansell SM, Infante JR, de Vos S, Hamlin PA, Kim SK, Whiting NC et al: Phase I dose-escalation study of SGN-75 in patients with CD70-positive relapsed/refractory non-Hodgkin lymphoma or metastatic renal cell carcinoma. Invest New Drugs 2014, 32(6):1246-1257.

50. Sehn LH, Herrera AF, Flowers CR, Kamdar MK, McMillan A, Hertzberg M, Assouline S, Kim TM, Kim WS, Ozcan M et al: Polatuzumab Vedotin in Relapsed or Refractory Diffuse Large B-Cell Lymphoma. J Clin Oncol 2020, 38(2):155-165.

51. Dornan D, Bennett F, Chen Y, Dennis M, Eaton D, Elkins K, French D, Go MA, Jack A, Junutula JR et al: Therapeutic potential of an anti-CD79b antibody-drug conjugate, anti-CD79b-vc-MMAE, for the treatment of non-Hodgkin lymphoma. Blood 2009, 114(13):2721-2729.

52. Buschow SI, van Balkom BW, Aalberts M, Heck AJ, Wauben M, Stoorvogel W: MHC class II-associated proteins in B-cell exosomes and potential functional implications for exosome biogenesis. Immunol Cell Biol 2010, 88(8):851-856.

53. Abrams T, Connor A, Fanton C, Cohen SB, Huber T, Miller K, Hong EE, Niu X, Kline J, Ison-Dugenny M et al: Preclinical Antitumor Activity of a Novel Anti-c-KIT Antibody-Drug Conjugate against Mutant and Wild-type c-KIT-Positive Solid Tumors. Clin Cancer Res 2018, 24(17):4297-4308.

54. Atay S, Banskota S, Crow J, Sethi G, Rink L, Godwin AK: Oncogenic KIT-containing exosomes increase gastrointestinal stromal tumor cell invasion. Proc Natl Acad Sci U S A 2014, 111(2):711-716.

55. Kovtun Y, Jones GE, Adams S, Harvey L, Audette CA, Wilhelm A, Bai C, Rui L, Laleau R, Liu F et al: A CD123-targeting antibody-drug conjugate, IMGN632, designed to eradicate AML while sparing normal bone marrow cells. Blood Adv 2018, 2(8):848-858.

56. Hong CS, Funk S, Muller L, Boyiadzis M, Whiteside TL: Isolation of biologically active and morphologically intact exosomes from plasma of patients with cancer. J Extracell Vesicles 2016, 5:29289.

57. Li F, Sutherland MK, Yu C, Walter RB, Westendorf L, Valliere-Douglass J, Pan L, Cronkite A, Sussman D, Klussman K et al: Characterization of SGN-CD123A, A Potent CD123-Directed Antibody-Drug Conjugate for Acute Myeloid Leukemia. Mol Cancer Ther 2018, 17(2):554-564.

58. de Bono JS, Concin N, Hong DS, Thistlethwaite FC, Machiels JP, Arkenau HT, Plummer R, Jones RH, Nielsen D, Windfeld K et al: Tisotumab vedotin in patients with advanced or metastatic solid tumours (InnovaTV 201): a first-in-human, multicentre, phase 1-2 trial. Lancet Oncol 2019, 20(3):383-393.

59. Che SPY, Park JY, Stokol T: Tissue Factor-Expressing Tumor-Derived Extracellular Vesicles Activate Quiescent Endothelial Cells via Protease-Activated Receptor-1. Front Oncol 2017, 7:261.

60. Garnier D, Magnus N, Lee TH, Bentley V, Meehan B, Milsom C, Montermini L, Kislinger T, Rak J: Cancer cells induced to express mesenchymal phenotype release exosome-like extracellular vesicles carrying tissue factor. J Biol Chem 2012, 287(52):43565-43572.

61. Herbener P, Schonfeld K, Konig M, Germer M, Przyborski JM, Bernoster K, Schuttrumpf J: Functional relevance of in vivo half antibody exchange of an IgG4 therapeutic antibody-drug conjugate. PLoS One 2018, 13(4):e0195823.

62. Welton JL, Khanna S, Giles PJ, Brennan P, Brewis IA, Staffurth J, Mason MD, Clayton A: Proteomics analysis of bladder cancer exosomes. Mol Cell Proteomics 2010, 9(6):1324-1338.

63. Purushothaman A, Bandari SK, Liu J, Mobley JA, Brown EE, Sanderson RD: Fibronectin on the Surface of Myeloma Cell-derived Exosomes Mediates Exosome-Cell Interactions. J Biol Chem 2016, 291(4):1652-1663.

64. O'Connor-McCourt M, Koropatnick J, Maleki S, Figueredo R, Tikhomirov I, Jaramillo M: Development of AVID100, a novel antibody–drug conjugate for the treatment of EGFR expressing solid tumors. European Journal of Cancer 2016, Volume 69, Supplement 1, S147.

65. Zhang H, Deng T, Liu R, Bai M, Zhou L, Wang X, Li S, Wang X, Yang H, Li J et al: Exosome-delivered EGFR regulates liver microenvironment to promote gastric cancer liver metastasis. Nat Commun 2017, 8:15016.

66. Goss GD, Vokes EE, Gordon MS, Gandhi L, Papadopoulos KP, Rasco DW, Fischer JS, Chu KL, Ames WW, Mittapalli RK et al: Efficacy and safety results of depatuxizumab mafodotin (ABT-414) in patients with advanced solid tumors likely to overexpress epidermal growth factor receptor. Cancer 2018, 124(10):2174-2183.

67. Choi DS, Choi DY, Hong BS, Jang SC, Kim DK, Lee J, Kim YK, Kim KP, Gho YS: Quantitative proteomics of extracellular vesicles derived from human primary and metastatic colorectal cancer cells. J Extracell Vesicles 2012, 1.

68. Setiady YY, Dong L, Skaletskaya A, Pinkas J, Lutz RJ, Lambert JM, Chittenden T: IMGN289, an EGFR-targeting antibody-drug conjugate, is effective against tumor cells that are resistant to EGFR tyrosine kinase inhibitors. In: Proceedings of the 105th Annual Meeting of the American Association for Cancer Research; 2014 Apr 5-9; San Diego, CA Philadelphia (PA): AACR; Cancer Res 2014;74(19 Suppl):Abstract nr 4513 2014.

69. Kharmate G, Hosseini-Beheshti E, Caradec J, Chin MY, Tomlinson Guns ES: Epidermal Growth Factor Receptor in Prostate Cancer Derived Exosomes. PLoS One 2016, 11(5):e0154967.

70. Xu R-h, Qiu M-Z, Zhang Y, Wei X-L, Hu C: First-in-human dose-escalation study of anti-EGFR ADC MRG003 in patients with relapsed/refractory solid tumors. ASCO 2020.

71. AbbVie`s pipeline hwaco-sph.

72. Al-Nedawi K, Meehan B, Kerbel RS, Allison AC, Rak J: Endothelial expression of autocrine VEGF upon the uptake of tumor-derived microvesicles containing oncogenic EGFR. Proc Natl Acad Sci U S A 2009, 106(10):3794-3799.

73. Ciravolo V, Huber V, Ghedini GC, Venturelli E, Bianchi F, Campiglio M, Morelli D, Villa A, Della Mina P, Menard S et al: Potential role of HER2-overexpressing exosomes in countering trastuzumab-based therapy. J Cell Physiol 2012, 227(2):658-667.

74. Rosenthal M, Curry R, Reardon DA, Rasmussen E, Upreti VV, Damore MA, Henary HA, Hill JS, Cloughesy T: Safety, tolerability, and pharmacokinetics of anti-EGFRvIII antibody-drug conjugate AMG 595 in patients with recurrent malignant glioma expressing EGFRvIII. Cancer Chemother Pharmacol 2019, 84(2):327-336.

75. Al-Nedawi K, Meehan B, Micallef J, Lhotak V, May L, Guha A, Rak J: Intercellular transfer of the oncogenic receptor EGFRvIII by microvesicles derived from tumour cells. Nat Cell Biol 2008, 10(5):619-624.

76. Damelin M, Bankovich A, Park A, Aguilar J, Anderson W, Santaguida M, Aujay M, Fong S, Khandke K, Pulito V et al: Anti-EFNA4 Calicheamicin Conjugates Effectively Target Triple-Negative Breast and Ovarian Tumor-Initiating Cells to Result in Sustained Tumor Regressions. Clin Cancer Res 2015, 21(18):4165-4173.

77. Gong J, Korner R, Gaitanos L, Klein R: Exosomes mediate cell contact-independent ephrin-Eph signaling during axon guidance. J Cell Biol 2016, 214(1):35-44.

78. Surguladze D, Pennello A, Ren X, Mack T, Rigby A, Balderes P, Navarro E, Amaladas N, Eastman S, Topper M et al: LY3076226, a novel anti-FGFR3 antibody drug conjugate exhibits potent and durable anti-tumor activity in tumor models harboring FGFR3 mutations or fusions. Cancer Res 2019;79(13 Suppl):Abstract nr 4835.

79. Saraswat M, Joenvaara S, Musante L, Peltoniemi H, Holthofer H, Renkonen R: N-linked (N-) Glycoproteomics of Urinary Exosomes. Mol Cell Proteomics 2015, 14(8):2298.

80. Nishigaki T, Takahashi T, Serada S, Fujimoto M, Ohkawara T, Hara H, Sugase T, Otsuru T, Saito Y, Tsujii S et al: Anti-glypican-1 antibody-drug conjugate is a potential therapy against pancreatic cancer. Br J Cancer 2020, 122(9):1333-1341.

81. Matsuzaki S, Serada S, Hiramatsu K, Nojima S, Matsuzaki S, Ueda Y, Ohkawara T, Mabuchi S, Fujimoto M, Morii E et al: Anti-glypican-1 antibody-drug conjugate exhibits potent preclinical antitumor activity against glypican-1 positive uterine cervical cancer. Int J Cancer 2018, 142(5):1056-1066.

82. Melo SA, Luecke LB, Kahlert C, Fernandez AF, Gammon ST, Kaye J, LeBleu VS, Mittendorf EA, Weitz J, Rahbari N et al: Glypican-1 identifies cancer exosomes and detects early pancreatic cancer. Nature 2015, 523(7559):177-182.

83. Pegram MD, Miles D, Tsui CK, Zong Y: HER2-Overexpressing/Amplified Breast Cancer as a Testing Ground for Antibody-Drug Conjugate Drug Development in Solid Tumors. Clin Cancer Res 2020, 26(4):775-786.

84. Park YH, Ahn HK, Kim J, Ahn JS, Im Y, Kim S, Lee S, Chung H, Park SJ: First-in-human phase I study of ALT-P7, a HER2-targeting antibody-drug conjugate in patients with HER2-positive advanced breast cancer. ASCO 2020.

85. Barok M, Le Joncour V, Martins A, Isola J, Salmikangas M, Laakkonen P, Joensuu H: ARX788, a novel anti-HER2 antibody-drug conjugate, shows anti-tumor effects in preclinical models of trastuzumab emtansine-resistant HER2-positive breast cancer and gastric cancer. Cancer Lett 2020, 473:156-163.

86. Andre F, Schartz NE, Movassagh M, Flament C, Pautier P, Morice P, Pomel C, Lhomme C, Escudier B, Le Chevalier T et al: Malignant effusions and immunogenic tumour-derived exosomes. Lancet 2002, 360(9329):295-305.

87. Lopez DM, Barve M, Wang J, Bullock AJ, Pectasides E, Vaishampayan U, Spira AI, Ulahannan S, Patnaik A, Sanborn RE et al: A phase I study of A166, a novel anti-HER2 antibody-drug conjugate (ADC), in patients with locally advanced/metastatic solid tumors. Mol Cancer Ther 2019;18(12 Suppl):Abstract nr B005.

88. Wang S, Xu F, Hong R, Xia W, Yu J, Tang W, Wei J, Song S, Wang Z, Zhang Z et al: BAT8001, a potent anti-HER2 antibody drug conjugate with a novel uncleavable linker to reduce toxicity for patients with HER2-positive tumor. Cancer Res 2019;79(13 Suppl):Abstract nr CT053 2019.

89. Li JY, Perry SR, Muniz-Medina V, Wang X, Wetzel LK, Rebelatto MC, Hinrichs MJ, Bezabeh BZ, Fleming RL, Dimasi N et al: A Biparatopic HER2-Targeting Antibody-Drug Conjugate Induces Tumor Regression in Primary Models Refractory to or Ineligible for HER2-Targeted Therapy. Cancer Cell 2016, 29(1):117-129.

90. Pernas S, Tolaney SM: HER2-positive breast cancer: new therapeutic frontiers and overcoming resistance. Ther Adv Med Oncol 2019, 11:1758835919833519.

91. Li J, Guo Y, Xue J, Peng W, Ge X, Zhao W, Dai C, Xue L, Tang W, Hu C: First-in-human phase I study of anti-HER2 ADC MRG002 in patients with relapsed/refractory solid tumors. ASCO 2020.

92. Sung MS, Hopf C, Upeslacis E, Golas J, Kaplan M, Khandke K, Charati M, Kotch F, Loganzo F, Geles K et al: NG-HER2 ADC (PF-06804103) is superior to trastuzumab emtansine in a mouse 'avatar' head-to-head clinical trial. Cancer Res 2018;78(13 Suppl):Abstract nr 818.

93. Le Joncour V, Martins A, Puhka M, Isola J, Salmikangas M, Laakkonen P, Joensuu H, Barok M: A Novel Anti-HER2 Antibody-Drug Conjugate XMT-1522 for HER2-Positive Breast and Gastric Cancers Resistant to Trastuzumab Emtansine. Mol Cancer Ther 2019, 18(10):1721-1730.

94. Rinnerthaler G, Gampenrieder SP, Greil R: HER2 Directed Antibody-Drug-Conjugates beyond T-DM1 in Breast Cancer. Int J Mol Sci 2019, 20(5).

95. Sheng X, Yan X, Wang L, Shi YX, Yao X, Luo H, Shi B, Liu JY, He Z, Yu G et al: Open-label, multicenter, phase 2 study of RC48-ADC, a HER2-targeting antibody-drug conjugate, in patients with locally advanced or metastatic urothelial carcinoma. Clin Cancer Res 2020.

96. Koga K, Matsumoto K, Akiyoshi T, Kubo M, Yamanaka N, Tasaki A, Nakashima H, Nakamura M, Kuroki S, Tanaka M et al: Purification, characterization and biological significance of tumor-derived exosomes. Anticancer Res 2005, 25(6A):3703-3707.

97. Hurvitz SA, Dirix L, Kocsis J, Bianchi GV, Lu J, Vinholes J, Guardino E, Song C, Tong B, Ng V et al: Phase II randomized study of trastuzumab emtansine versus trastuzumab plus docetaxel in patients with human epidermal growth factor receptor 2-positive metastatic breast cancer. J Clin Oncol 2013, 31(9):1157-1163.

98. Barok M, Joensuu H, Isola J: Trastuzumab emtansine: mechanisms of action and drug resistance. Breast Cancer Res 2014, 16(2):209.

99. Battke C, Ruiss R, Welsch U, Wimberger P, Lang S, Jochum S, Zeidler R: Tumour exosomes inhibit binding of tumour-reactive antibodies to tumour cells and reduce ADCC. Cancer Immunol Immunother 2011, 60(5):639-648.

100. Ogitani Y, Aida T, Hagihara K, Yamaguchi J, Ishii C, Harada N, Soma M, Okamoto H, Oitate M, Arakawa S et al: DS-8201a, A Novel HER2-Targeting ADC with a Novel DNA Topoisomerase I Inhibitor, Demonstrates a Promising Antitumor Efficacy with Differentiation from T-DM1. Clin Cancer Res 2016, 22(20):5097-5108.

101. Clayton A, Mitchell JP, Court J, Mason MD, Tabi Z: Human tumor-derived exosomes selectively impair lymphocyte responses to interleukin-2. Cancer Res 2007, 67(15):7458-7466.

102. Dokter W, Ubink R, van der Lee M, van der Vleuten M, van Achterberg T, Jacobs D, Loosveld E, van den Dobbelsteen D, Egging D, Mattaar E et al: Preclinical profile of the HER2-targeting ADC SYD983/SYD985: introduction of a new duocarmycin-based linker-drug platform. Mol Cancer Ther 2014, 13(11):2618-2629.

103. Barok M, Puhka M, Vereb G, Szollosi J, Isola J, Joensuu H: Cancer-derived exosomes from HER2-positive cancer cells carry trastuzumab-emtansine into cancer cells leading to growth inhibition and caspase activation. BMC Cancer 2018, 18(1):504.

104. Raab-Westphal S, Marshall JF, Goodman SL: Integrins as Therapeutic Targets: Successes and Cancers. Cancers (Basel) 2017, 9(9).

105. Bendell J, Moore K, Qin A, Johnson D, Schindler J, Papadopoulos K, Tolcher AW: A phase I study of IMGN388, an antibody drug conjugate targeting av integrin, in patients with solid tumors. European Journal of Cancer Supplements 2010, 8(7):152.

106. Hoshino A, Costa-Silva B, Shen TL, Rodrigues G, Hashimoto A, Tesic Mark M, Molina H, Kohsaka S, Di Giannatale A, Ceder S et al: Tumour exosome integrins determine organotropic metastasis. Nature 2015, 527(7578):329-335.

107. Calvet L, Lefebvre A, Nicolazzi C, Blot L, Thomas C, Baudat Y, Cameron B, Garcia-Echeverria C, Mayaux J, Blanc V et al: Outstanding preclinical efficacy of a novel maytansinoid-antibody-drug conjugate targeting LAMP1 in patient-derived xenograft solid tumors. In: Proceedings of the 107th Annual Meeting of the American Association for Cancer Research; 2016 Apr 16-20; New Orleans, LA Philadelphia (PA): AACR; Cancer Res 2016;76(14 Suppl):Abstract nr 1197 2016.

108. Hassan R, Blumenschein GR, Jr., Moore KN, Santin AD, Kindler HL, Nemunaitis JJ, Seward SM, Thomas A, Kim SK, Rajagopalan P et al: First-in-Human, Multicenter, Phase I Dose-Escalation and Expansion Study of Anti-Mesothelin Antibody-Drug Conjugate Anetumab Ravtansine in Advanced or Metastatic Solid Tumors. J Clin Oncol 2020:JCO1902085.

109. Quanz M, Hagemann UB, Zitzmann-Kolbe S, Stelte-Ludwig B, Golfier S, Elbi C, Mumberg D, Ziegelbauer K, Schatz CA: Anetumab ravtansine inhibits tumor growth and shows additive effect in combination with targeted agents and chemotherapy in mesothelin-expressing human ovarian cancer models. Oncotarget 2018, 9(75):34103-34121.

110. Clarke J, Chu SC, Siu LL, Machiels JP, Markman B, Heinhuis K, Millward M, Lolkema M, Patel SP, de Souza P et al: BMS-986148, an anti-mesothelin antibody-drug conjugate (ADC), alone or in combination with nivolumab demonstrates clinical activity in patients with select advanced solid tumors. Mol Cancer Ther 2019;18(12 Suppl):Abstract nr B057.

111. Weekes CD, Lamberts LE, Borad MJ, Voortman J, McWilliams RR, Diamond JR, de Vries EG, Verheul HM, Lieu CH, Kim GP et al: Phase I Study of DMOT4039A, an Antibody-Drug Conjugate Targeting Mesothelin, in Patients with Unresectable Pancreatic or Platinum-Resistant Ovarian Cancer. Mol Cancer Ther 2016, 15(3):439-447.

112. Cho S, Zammarchi F, Williams DG, Havenith CEG, Monks NR, Tyrer P, D'Hooge F, Fleming R, Vashisht K, Dimasi N et al: Antitumor Activity of MEDI3726 (ADCT-401), a Pyrrolobenzodiazepine Antibody-Drug Conjugate Targeting PSMA, in Preclinical Models of Prostate Cancer. Mol Cancer Ther 2018, 17(10):2176-2186.

113. Mizutani K, Terazawa R, Kameyama K, Kato T, Horie K, Tsuchiya T, Seike K, Ehara H, Fujita Y, Kawakami K et al: Isolation of prostate cancer-related exosomes. Anticancer Res 2014, 34(7):3419-3423.

114. Galsky MD, Eisenberger M, Moore-Cooper S, Kelly WK, Slovin SF, DeLaCruz A, Lee Y, Webb IJ, Scher HI: Phase I trial of the prostate-specific membrane antigen-directed immunoconjugate MLN2704 in patients with progressive metastatic castration-resistant prostate cancer. J Clin Oncol 2008, 26(13):2147-2154.

115. Petrylak DP, Kantoff P, Vogelzang NJ, Mega A, Fleming MT, Stephenson JJ, Jr., Frank R, Shore ND, Dreicer R, McClay EF et al: Phase 1 study of PSMA ADC, an antibody-drug conjugate targeting prostate-specific membrane antigen, in chemotherapy-refractory prostate cancer. Prostate 2019, 79(6):604-613.

116. Liu T, Mendes DE, Berkman CE: Functional prostate-specific membrane antigen is enriched in exosomes from prostate cancer cells. Int J Oncol 2014, 44(3):918-922.

117. Tang W, Huang X, Ou Z, Yan H, Gan J, Dong Q, Tan B, Yang Y, Guo Y, Li S et al: BAT8003, a potent anti-Trop-2 antibody-drug conjugate, for the treatment of triple negative breast cancer. Cancer Res 2019;79(4 Suppl):Abstract nr P6-20-16.

118. Trerotola M, Ganguly KK, Fazli L, Fedele C, Lu H, Dutta A, Liu Q, De Angelis T, Riddell LW, Riobo NA et al: Trop-2 is up-regulated in invasive prostate cancer and displaces FAK from focal contacts. Oncotarget 2015, 6(16):14318-14328.

119. Okajima D, Yamaguchi J, Kitamura M, Kamei R, Maejima T, Shibutani T, Yasuda S, Toki T, Karibe T, Fujitani T et al: DS-1062a, a novel TROP2-targeting antibody-drug conjugate with a novel DNA topoisomerase I inhibitor DXd, demonstrates potent antitumor activity in preclinical models. Mol Cancer Ther 2019;18(12 Suppl):Abstract nr C026.

120. King GT, Eaton KD, Beagle BR, Zopf CJ, Wong GY, Krupka HI, Hua SY, Messersmith WA, El-Khoueiry AB: A phase 1, dose-escalation study of PF-06664178, an anti-Trop-2/Aur0101 antibody-drug conjugate in patients with advanced or metastatic solid tumors. Invest New Drugs 2018, 36(5):836-847.

121. Cardillo TM, Govindan SV, Sharkey RM, Trisal P, Goldenberg DM: Humanized anti-Trop-2 IgG-SN-38 conjugate for effective treatment of diverse epithelial cancers: preclinical studies in human cancer xenograft models and monkeys. Clin Cancer Res 2011, 17(10):3157-3169.

122. Bardia A, Mayer IA, Vahdat LT, Tolaney SM, Isakoff SJ, Diamond JR, O'Shaughnessy J, Moroose RL, Santin AD, Abramson VG et al: Sacituzumab Govitecan-hziy in Refractory Metastatic Triple-Negative Breast Cancer. N Engl J Med 2019, 380(8):741-751.

123. Liu Y, Lian W, Zhao X, Diao Y, Xu J, Xiao L, Qing Y, Xue T, Wang J: A first-in-human study of SKB264 in patients with locally advanced unresectable/metastatic solid tumors who are refractory to available standard therapies. ASCO 2020 2020.

124. Goldenberg DM, Stein R, Sharkey RM: The emergence of trophoblast cell-surface antigen 2 (TROP-2) as a novel cancer target. Oncotarget 2018, 9(48):28989-29006.
