# Supplementary material for: Extracellular vesicles as modifiers of antibody‐drug conjugate efficacy
Source: J Extracell Vesicles. 2021 Feb 13;10(4):e12070. doi: 10.1002/jev2.12070 (PMC7881363; doi:10.1002/jev2.12070)
Supplement: Supplementary file 2 — Supplementary Table 2: Trastuzumab‐based anti‐HER2 antibody‐drug conjugates [file JEV2-10-e12070-s002.docx]

| **Antibody-drug conjugate**  **/ Developer** | **Monoclonal antibody** | **Indication (stage)** |
| --- | --- | --- |
| ADCT-502 (ADC Therapeutics) [1] | Trastuzumab | HER2+ solid tumors (phase I) discontinued |
| ALT-P7 (Alteogen) [2] | Trastuzumab biobetter | HER2+ breast cancer (phase I) |
| A166 (Klus Pharma) [3] | Same amino acid sequence as in trastuzumab | HER2+ solid tumors (phase I) |
| BAT8001 (Bio-Thera Solutions) [4] | Trastuzumab | HER2+ advanced breast cancer (phase III) |
| PF-06804103 (Pfizer) [5] | Engineered cysteine mutant  variant of trastuzumab | HER2+ breast cancer, and gastric cancer (phase I) |
| Trastuzumab deruxtecan (Daiichi Sankyo) [6] | Trastuzumab | Approved for  HER2+ advanced breast cancer |
| Trastuzumab duocarmazine (Synthon) [7] | Trastuzumab | HER2+ advanced breast cancer, and metastatic endometrial cancer (phase III) |
| Trastuzumab emtansine (Genentech/Roche) [8, 9] | Trastuzumab | Approved for   1. HER2+ advanced breast cancer 2. HER2+ early breast cancer |

**Supplementary Table 2. Trastuzumab-based anti-HER2 antibody-drug conjugates**

**Abbreviation**

HER2: Human epidermal growth factor receptor-2

**References**

1. Pegram MD, Miles D, Tsui CK, Zong Y: HER2-Overexpressing/Amplified Breast Cancer as a Testing Ground for Antibody-Drug Conjugate Drug Development in Solid Tumors. Clin Cancer Res 2020, 26(4):775-786.

2. Park YH, Ahn HK, Kim J, Ahn JS, Im Y, Kim S, Lee S, Chung H, Park SJ: First-in-human phase I study of ALT-P7, a HER2-targeting antibody-drug conjugate in patients with HER2-positive advanced breast cancer. ASCO 2020.

3. Lopez DM, Barve M, Wang J, Bullock AJ, Pectasides E, Vaishampayan U, Spira AI, Ulahannan S, Patnaik A, Sanborn RE et al: A phase I study of A166, a novel anti-HER2 antibody-drug conjugate (ADC), in patients with locally advanced/metastatic solid tumors. Mol Cancer Ther 2019;18(12 Suppl):Abstract nr B005.

4. Wang S, Xu F, Hong R, Xia W, Yu J, Tang W, Wei J, Song S, Wang Z, Zhang Z et al: BAT8001, a potent anti-HER2 antibody drug conjugate with a novel uncleavable linker to reduce toxicity for patients with HER2-positive tumor. Cancer Res 2019;79(13 Suppl):Abstract nr CT053 2019.

5. Graziani EI, Sung M, Ma D, Narayanan B, Marquette K, Puthenveetil S, Tumey LN, Bikker J, Casavant J, Bennett EM et al: PF-06804103, a site-specific anti-HER2 antibody-drug conjugate for the treatment of HER2-expressing breast, gastric, and lung cancers. Mol Cancer Ther 2020.

6. Ogitani Y, Aida T, Hagihara K, Yamaguchi J, Ishii C, Harada N, Soma M, Okamoto H, Oitate M, Arakawa S et al: DS-8201a, A Novel HER2-Targeting ADC with a Novel DNA Topoisomerase I Inhibitor, Demonstrates a Promising Antitumor Efficacy with Differentiation from T-DM1. Clin Cancer Res 2016, 22(20):5097-5108.

7. Dokter W, Ubink R, van der Lee M, van der Vleuten M, van Achterberg T, Jacobs D, Loosveld E, van den Dobbelsteen D, Egging D, Mattaar E et al: Preclinical profile of the HER2-targeting ADC SYD983/SYD985: introduction of a new duocarmycin-based linker-drug platform. Mol Cancer Ther 2014, 13(11):2618-2629.

8. Hurvitz SA, Dirix L, Kocsis J, Bianchi GV, Lu J, Vinholes J, Guardino E, Song C, Tong B, Ng V et al: Phase II randomized study of trastuzumab emtansine versus trastuzumab plus docetaxel in patients with human epidermal growth factor receptor 2-positive metastatic breast cancer. J Clin Oncol 2013, 31(9):1157-1163.

9. Barok M, Joensuu H, Isola J: Trastuzumab emtansine: mechanisms of action and drug resistance. Breast Cancer Res 2014, 16(2):209.
